# Discovery of an RmlC/D fusion protein in the microalga *Prymnesium parvum* and its implications for NDP-β-l-rhamnose biosynthesis in microalgae

**DOI:** 10.1074/jbc.RA118.006440

**Published:** 2019-04-22

**Authors:** Ben A. Wagstaff, Martin Rejzek, Sakonwan Kuhaudomlarp, Lionel Hill, Ilaria Mascia, Sergey A. Nepogodiev, Helge C. Dorfmueller, Robert A. Field

**Affiliations:** From the ‡ Department of Biological Chemistry, John Innes Centre, Norwich Research Park, Norwich, NR4 7UH, United Kingdom,; § Division of Molecular Microbiology, School of Life Sciences, University of Dundee, Dundee, DD1 5EH, United Kingdom, and; ¶ Université Grenoble Alpes, CNRS, CERMAV, 38000, Grenoble, France

**Keywords:** algae, carbohydrate biosynthesis, bioinformatics, carbohydrate processing, glycobiology, enzyme, pathway evolution, Prymnesium parvum, rhamnose, sugar nucleotides

## Abstract

The 6-deoxy sugar l-rhamnose (l-Rha) is found widely in plant and microbial polysaccharides and natural products. The importance of this and related compounds in host–pathogen interactions often means that l-Rha plays an essential role in many organisms. l-Rha is most commonly biosynthesized as the activated sugar nucleotide uridine 5′-diphospho-β-l-rhamnose (UDP-β-l-Rha) or thymidine 5′-diphospho-β-l-rhamnose (TDP-β-l-Rha). Enzymes involved in the biosynthesis of these sugar nucleotides have been studied in some detail in bacteria and plants, but the activated form of l-Rha and the corresponding biosynthetic enzymes have yet to be explored in algae. Here, using sugar-nucleotide profiling in two representative algae, *Euglena gracilis* and the toxin-producing microalga *Prymnesium parvum*, we show that levels of UDP- and TDP-activated l-Rha differ significantly between these two algal species. Using bioinformatics and biochemical methods, we identified and characterized a fusion of the RmlC and RmlD proteins, two bacteria-like enzymes involved in TDP-β-l-Rha biosynthesis, from *P. parvum*. Using this new sequence and also others, we explored l-Rha biosynthesis among algae, finding that although most algae contain sequences orthologous to plant-like l-Rha biosynthesis machineries, instances of the RmlC-RmlD fusion protein identified here exist across the Haptophyta and Gymnodiniaceae families of microalgae. On the basis of these findings, we propose potential routes for the evolution of nucleoside diphosphate β-l-Rha (NDP-β-l-Rha) pathways among algae.

## Introduction

The 6-deoxy sugar rhamnose (Rha)^3^ is found in glycoproteins, structural polysaccharides, and natural products across the microbes, algae, and plants, but it is rarely found in animals (1). Most commonly found as the l enantiomer, l-Rha is present in the capsules or cell walls of bacteria (2, 3), fungi (4), and plants (5); it is also present in the lesser-studied glycans of viruses (6). Previous work has found l-Rha in fungal and bacterial glycans that play crucial roles in host–pathogen interactions (7, 8). Because of the rarity of l-Rha biosynthetic capability in animals, and the essentiality of this sugar for the virulence of numerous pathogens, the microbial biosynthetic pathway for l-Rha production has drawn interest as a potential antimicrobial drug target (9, 10).

**Figure 1.**
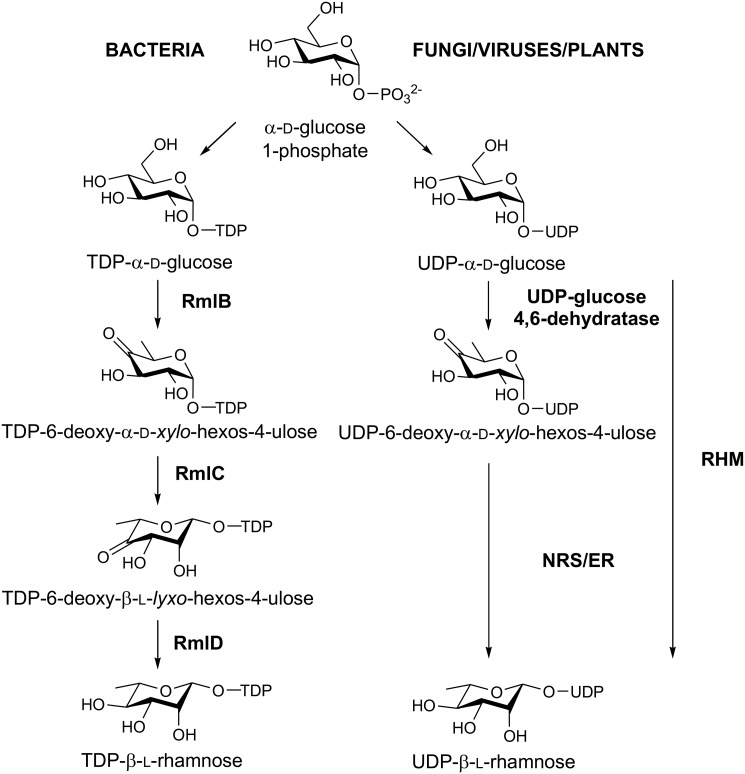
**Biosynthesis of NDP-β-l-Rha in bacteria, fungi, viruses and plants.** In bacteria, three independent enzymes catalyze dehydration, epimerization, and reduction steps to yield TDP-β-l-Rha—RmlB, RmlC, and RmlD, respectively. In plants, fungi, and viruses these three enzymatic activities are found on multi-functional enzymes. NRS/ER from *Arabidopsis thaliana* has been shown to be bifunctional and contains both 3,5-epimerase and 4-reductase activities. RHM proteins from *A. thaliana* have been shown to catalyze both of the previous steps, including the initial 4,6-dehydration step to form the keto-sugar.

The activated l-Rha species for carbohydrate polymer biosynthesis are thymidine 5′-diphospho-β-l-rhamnose (TDP-β-l-Rha) and uridine 5′-diphospho-β-l-rhamnose (UDP-β-l-Rha), which are produced biosynthetically from thymidine 5′-diphospho-α-d-glucose (TDP-α-d-Glc) and uridine 5′-diphospho-α-d-glucose (UDP-α-d-Glc), respectively (Fig. 1). In bacteria, many examples have shown that TDP-β-l-Rha is produced from TDP-α-d-Glc by the action of three independent enzymes (11): RmlB (a 4,6-dehydratase) (PDB entry 1G1A), RmlC (a 3,5-epimerase) (PDB entry 2IXJ), and RmlD (a 4-reductase) (PDB entries 1KBZ and 4WPG). Beyond biopolymer production, RmlB is a central player in natural products biosynthesis; its product, TDP-6-deoxy-α-d-*xylo*-hexos-4-ulose (see IUPAC-IUBMB nomenclature in Ref. 12), is subject to numerous enzymatic processes that produce diverse sugar nucleotides that serve as substrates for natural product “glycodiversification” (13, 14). The Rml enzymes B–D, have been studied in some detail, with crystal structures of all three having been solved (15–17, 55). More recently, enzymes from plants (18, 19), fungi (8), and even viruses (20) have been shown to synthesize UDP-β-l-Rha from UDP-α-d-Glc. These enzymes are structurally distinct from their bacterial counterparts, with multiple enzymatic activities found in individual proteins. For plants, the dehydratase, epimerase, and reductase activities (Fig. 1) are often found on one large protein (RHM) (18), but there are also instances of the three enzymatic activities being shared over two proteins; a UDP-α-d-Glc 4,6-dehydratase and a bifunctional nucleotide–rhamnose synthase/epimerase-reductase (NRS/ER) (19). Fungi and viruses also contain orthologs of the bifunctional epimerase-reductase NRS/ER plant system, often showing substrate specificity for the uridine diphospho sugars over their thymidine counterparts (8, 20). Nonetheless, deciphering the *in vitro* nucleotide specificity of the bacterial and plant-like enzymes has remained a challenge because of either poor expression of the recombinant enzymes or the instability of enzyme substrates, particularly TDP- and UDP-6-deoxy-α-d-*xylo*-hexos-4-ulose, as discussed by Han *et al.* (19). However, it is generally accepted that the Rml enzymes favor TDP-activated substrates over their UDP counterparts (1, 21), although plant NRS/ER or RHM enzymes favor the UDP-based substrates (8, 18, 20).

Although the biosynthesis of l-Rha has been studied in some detail in bacteria, fungi, and plants, there is little information regarding the diverse algal groups, even though the presence of l-Rha has been noted in structural polysaccharides of macroalgae (22), and in the surface glycans and pellicle of the green microalga *Euglena gracilis* (23–25). Recent work by O'Neill *et al.* (26) identified prospective rhamnoside hydrolase genes in *E. gracilis*, but did not explore the associated biochemical events in any detail. Evolutionarily distinct algae derived from the red algal plastid have also been found to contain l-Rha, with early work identifying the sugar in cell preparations from the haptophytes *Isochrysis galbana* and *Prymnesium parvum* (27). Despite the reported occurrence of l-Rha in the algae, it is not known how they produce this sugar, which nucleotides they use to activate l-Rha, or how and where algae acquired their l-Rha biosynthetic machinery in evolutionary terms.

By profiling intracellular sugar nucleotides of a representative euglenid, *E. gracilis*, and a haptophyte, *P. parvum*, we first show that *E. gracilis* contains primarily UDP-β-l-rhamnose whereas *P. parvum* contains primarily TDP-β-l-rhamnose. We then show that *E. gracilis* contains sequences orthologous to plant-like NRS/ER whereas *P. parvum* contains a novel chimeric version of bacterial RmlC and RmlD (referred to hereafter as RmlCD). We go on to biochemically characterize a recombinant form of this RmlCD chimera and show that it produces both TDP-β-l-rhamnose and UDP-β-l-rhamnose from TDP- and UDP-6-deoxy-α-d-*xylo*-hexos-4-ulose, respectively. Using these new gene sequences, we explore the diversity of NDP-β-l-rhamnose biosynthetic pathways across the algal taxonomic groups. We show that plant-like NRS/ER or RHM sequences are widespread among algae, whereas occurrences of the RmlCD chimera seen in *P. parvum* can be found primarily in the Haptophyta and Gymnodiniaceae families. Using these findings, we evaluate potential routes for the evolution of nucleoside diphosphate β-l-Rha (NDP-β-l-Rha) pathways among algae.

## Results

### Sugar-nucleotide profiling

**Scheme 1.**
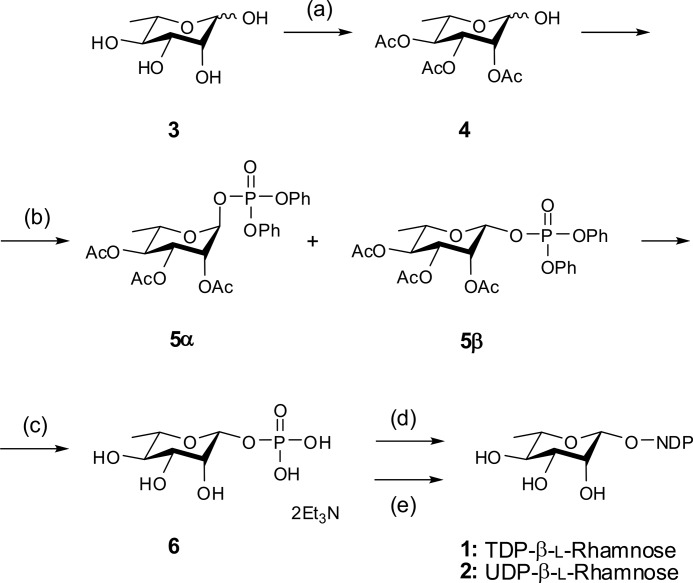
**Synthetic approach to TDP-β-l-Rha and UDP-β-l-Rha.** Reagents and conditions: *a*, (i) Ac_2_O, HBr-AcOH (33%), 0 to 20 °C, 2.5 h, (ii) AcONa, (iii) ice-water, 1 h, 29%. *b*, (i) Cl(O)P(OPh)_2_, DMAP, CH_2_Cl_2_, rt; (ii) silica gel column chromatography hexane-ethyl acetate-Et_3_N, 5α 6%, 5β 49%. *c*, (i) 5β only, H_2_, PtO_2_, AcOEt-EtOH; (ii) MeOH-Et_3_N-H_2_O 2:1:1, 4 °C, 7 d, 83% over two steps. *d*, TMP-morpholidate, pyridine, 4 °C, 5 d, LH20 and RP C18 purification, 16%. *e*, UMP-morpholidate, pyridine, 4 °C, 7 d, LH20 and RP C18 purification, 21%.

We first sought to investigate the preferences for TDP-β-l-Rha or UDP-β-l-Rha in the euglenid *E. gracilis* and compare it to that of the haptophyte *P. parvum*. We also looked at the presence of TDP- and/or UDP-α-d-Glc, the likely biosynthetic precursors of the corresponding l-Rha derivatives. To underpin these studies, authentic standards of TDP-β-l-Rha and UDP-β-l-Rha were required. Because of inconsistencies in the published literature on the chemical synthesis of these compounds, we conducted a thorough re-examination of published methods and product characterization and produced both sugar nucleotides unambiguously. The synthetic scheme employed is shown in Scheme 1; full details of this chemical synthesis can be found in the supporting material.

### Quantification of intracellular levels of NDP-β-l-Rha in algal cells

**Figure 2.**
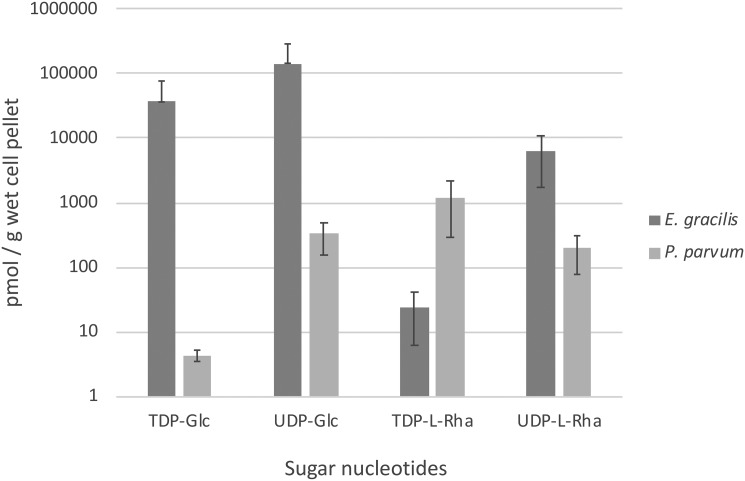
**Assessment of levels of TDP- or UDP-activated glucose and l-Rha in *E. gracilis* and *P. parvum*.** *Error bars* represent S.D. of three biological repeats. S.D. values for *E. gracilis* (TDP-Glc and UDP-Glc data points) were greater than the average and so only positive S.D. values could be plotted on the logarithmic graph.

Axenic cultures of *E. gracilis* and *P. parvum* were grown and harvested between mid- to late-log phase and at the same time of day to avoid differences in sugar-nucleotide levels because of the differences in growth phase. For *E. gracilis*, this represented ∼6 days of growth, whereas for *P. parvum* late-log phase was usually achieved after ∼14 days of growth. Cold ethanol was used to bring about cell lysis and to extract the target metabolites under mild conditions (28), thus minimizing degradation of the labile sugar nucleotides. In addition, ethanol efficiently precipitates and inactivates cytosolic enzymes and prevents undesired enzymatic degradation. After partitioning between water and butan-1-ol, the aqueous layers were then subjected to solid phase extraction using ENVICarb graphitized carbon column (29). This method was previously shown to have extraction recoveries ranging from 68 to 100%. Based on previous work by Pabst and co-workers (30), an LC-MS/MS method was used to analyze and quantify the intracellular sugar nucleotides. A surface-conditioned porous graphitic carbon column (Hypercarb) was used for separation and Xevo TQ-S tandem quadrupole mass spectrometer operated in multiple reaction monitoring (MRM) mode was used to detect the target analytes. Authentic standards of sugar nucleotides were used to generate MRM transitions and to determine retention times (Table S1). When in doubt, co-injection of samples with standards was used to further confirm analyte identification. Internal standards (guanosine 5′-diphospho-α-d-glucose (GDP-α-d-Glc) for *P. parvum*, uridine 5′-diphospho-2-acetamido-2-deoxy-α-d-glucuronic acid (UDP-α-d-GlcNAcA) for *E. gracilis*) were used for data normalization allowing direct comparison of relative sugar nucleotide levels between *E. gracilis* and *P. parvum* (Fig. 2).

LC-MS/MS results from biological triplicate show target NDP sugars ranging from low picomole to mid nanomole levels per gram of wet algal cell pellet (Fig. 2). *E. gracilis* contains ∼4-fold more UDP-α-d-Glc than TDP-α-d-Glc, at the mid nanomole range. Although levels of both TDP-α-d-Glc and UDP-α-d-Glc were lower in *P. parvum*, at the low to high picomole range, levels of TDP-α-d-Glc were significantly lower, with UDP-α-d-Glc ∼82-fold more abundant than TDP-α-d-Glc in *P. parvum*. These results appeared to have little correlation with the levels of activated l-Rha. *E. gracilis* contained ∼260 times more UDP-β-l-Rha than TDP-β-l-Rha and conversely *P. parvum* contained almost 6 times more TDP-β-l-Rha than UDP-β-l-Rha. Both organisms contained appreciable levels of both activated forms of l-Rha ranging from 24 pmol to 6.3 nmol/g pellet. These results suggest that *E. gracilis* likely contains a plant-like l-Rha biosynthesis pathway, whereas *P. parvum* may contain a bacteria-like l-Rha biosynthesis pathway. The presence of both forms of activated l-Rha in *P. parvum*, in particular, suggested multiple biosynthetic pathways or enzyme promiscuity.

### Identification of l-Rha biosynthetic genes in E. gracilis and P. parvum

To identify transcripts from *E. gracilis* and *P. parvum* involved in NDP-β-l-Rha biosynthesis, BLASTp searches were carried out against a transcriptome of *E. gracilis* that we recently reported on (31) and a publicly available transcriptome of *P. parvum* (Texoma1, Marine Microbial Eukaryote Transcriptome Sequencing Project). Query sequences used in the BLASTp analysis were RmlC (NP_217982.1), NRS/ER (NP_564806.1), and RHM (NP_177978.1). We found that *E. gracilis* contained a transcript orthologous to NRS/ER (32.3% sequence identity) whereas *P. parvum* contained a sequence more similar to RmlC at the N terminus of the protein (36.8% sequence identity) and to RmlD (NP_217783.1) at the C terminus of the protein sequence (35.5% sequence identity). These results agree with the above sugar-nucleotide profiling results and suggest that *E. gracilis* contains a plant-like NRS/ER biosynthetic pathway producing primarily UDP-β-l-Rha, whereas *P. parvum* contains a bacterial RmlCD chimera producing primarily TDP-β-l-Rha. The lack of an NRS/ER homolog in *P. parvum* combined with the observation that this organism also contains appreciable UDP-β-l-Rha suggested either a novel UDP-β-l-Rha biosynthetic pathway, or that the RmlCD chimera was capable of producing both TDP-β-l-Rha and UDP-β-l-Rha *in vivo*.

### Recombinant expression and biochemical characterization of P. parvum RmlCD fusion protein synthesizing TDP-β-l-Rha and UDP-β-l-Rha

To confirm the activity of the newly discovered *P. parvum* RmlCD chimera (CAMPEP_0191228776) as a bifunctional 3,5-epimerase/4-reductase producing NDP-β-l-Rha, we next sought to clone and biochemically characterize the protein. The recombinant protein originating from *P. parvum* was produced by heterologous expression in *Escherichia coli*. The sequence was codon optimized for expression in *E. coli* and cloned into the pOPINF vector (32). The recombinant plasmid containing the putative *P. parvum* RmlCD fusion sequence was expressed in *E. coli* SoluBL21^TM^ (Genlantis) cells. Using this methodology, reasonable levels of pure protein could be obtained (4 mg liter^−1^ *E. coli* culture as determined by Bradford assay) (Fig. S2).

**Figure 3.**
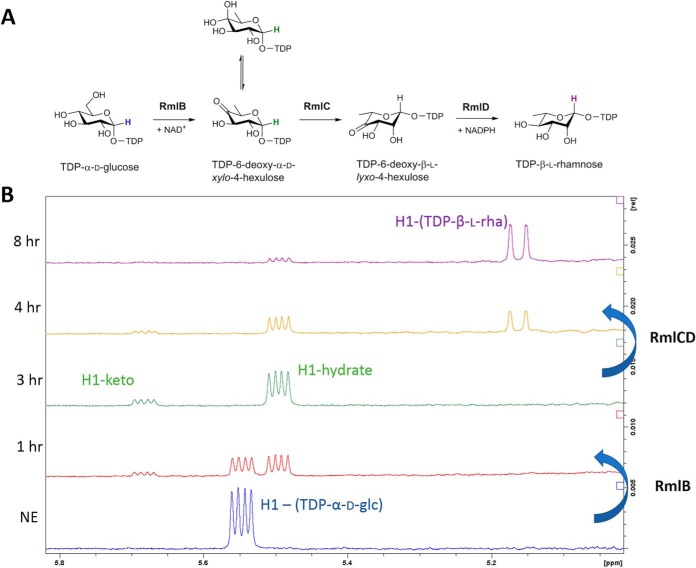
**Biochemical characterization of a RmlCD fusion protein from *P. parvum*.** *A*, general reaction scheme carried out by RmlB, RmlC, and RmlD. *B*, ^1^H NMR analysis following the reaction of RmlB from *S. enterica* Typhimurium and RmlCD from *P. parvum* forming TDP-β-l-Rha from TDP-α-d-Glc. Signals corresponding to the anomeric proton of TDP-6-deoxy-α-d-*xylo*-hexos-4-ulose (keto, 5.68 ppm; hydrate, 5.48 pm) can be observed 1 h after addition of *S. enterica* Typhimurium RmlB. After 3 h, *P. parvum* RmlCD is added and subsequent formation of TDP-β-l-Rha can be observed corresponding to the loss of signals at 5.48 and 5.68 ppm and formation of a new signal at 5.16 ppm corresponding to the anomeric proton signal of TDP-β-l-Rha. The reaction is seen to have gone to almost 80% (as judged by peak area integration) by 8 h to 4 h after addition of RmlCD.

For biochemical characterization of *P. parvum* RmlCD, a combination of ^1^H NMR and ESI-MS were used. Based on the known activities of the individual enzymes, the enzyme was anticipated to produce TDP-β-l-Rha starting from TDP-6-deoxy-α-d-*xylo*-hexos-4-ulose (Fig. 3*A*). TDP-6-deoxy-α-d-*xylo*-hexos-4-ulose was produced enzymatically using RmlB from *Salmonella enterica* serovar Typhimurium (*S. enterica* Typhimurium) (33) and TDP-α-d-glucose in a buffer containing the cofactors MgCl_2_, NAD^+^, and NADPH (required cofactor for RmlCD). The reaction was judged to have gone to completion when the anomeric proton signal of TDP-α-d-glucose disappeared, as observed by ^1^H NMR (Fig. 3*B*). At this point, *P. parvum* RmlCD was added to the reaction mixture and the reaction was followed by ^1^H NMR, whereby formation of the anomeric signal of TDP-β-l-Rha could be easily followed (Fig. 3*B*). ESI-MS of the reaction mixture confirmed the presence of the expected mass for TDP-β-l-Rha (547.0737, 0.2 ppm).

To evaluate the ability of RmlCD to produce UDP-β-l-Rha, UDP-6-deoxy-α-d-*xylo*-hexos-4-ulose was required. This compound is not commercially available and it could not be produced using *S. enterica* Typhimurium RmlB. We therefore employed recombinant viral enzyme ATCV-1 UDP-glucose 4,6-dehydratase, (Fig. S3*A*). Using identical reaction conditions to those with RmlB, we produced UDP-6-deoxy-α-d-*xylo*-hexos-4-ulose using ATCV-1 UDP-glucose 4,6-dehydratase and upon reaction completion we added RmlCD (Fig. S3*B*). Using this methodology, we were able to monitor the formation of UDP-β-l-Rha at a similar rate to that of TDP-β-l-Rha (Fig. S3*C*) suggesting that RmlCD is likely responsible for production of both TDP-β-l-Rha and UDP-β-l-Rha seen in *P. parvum* in the earlier sugar-nucleotide profiling results.

### Distribution of l-Rha biosynthetic genes in algae

**Figure 4.**
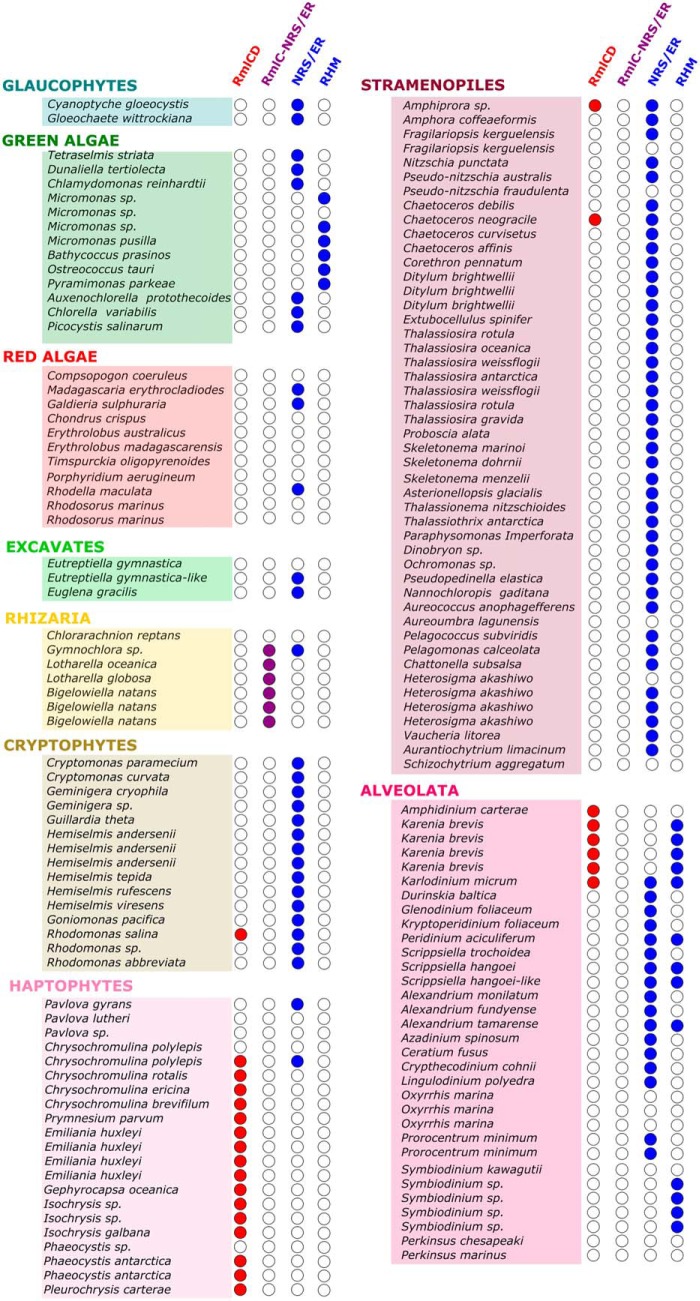
**Table showing the distribution of l-Rha biosynthesis genes in algae.** A total of 151 transcriptomes or genomes were analyzed for the presence of NDP-β-l-Rha biosynthetic genes from bacteria-like (RmlCD) or plant-like (NRS/ER or RHM) pathways. Where a transcript was identified for a given gene, a *filled circle* can be found. For bacteria-like pathways (*i.e.* RmlCD chimera) *circles* are *filled red*. For plant-like pathways (*i.e.* NRS/ER or RHM) *circles* are *filled blue*. When a fusion of bacterial RmlC and plant NRS/ER is observed, *circles* are *filled purple*. Multiple mention of the same species name means that different strains have been analyzed. For a full list of transcriptome, genome, and corresponding sequence identifiers, along with strains used in this study, refer to Table S2.

To evaluate the distribution of the NRS/ER, RHM, and RmlCD pathways among algae, BLAST searches were performed using reference sequences from bacteria and plants that were known to be involved in these pathways. BLASTp searches were carried out against the putative proteins assembled and translated from the algal transcriptomes and genomes using protein sequences for NRS/ER (NP_564806.1), RHM (NP_177978.1), and the newly discovered RmlCD chimera from *P. parvum* (CAMPEP_0191228776). We found that in most instances the bacterial pathway (represented by RmlCD) and the plant pathway (represented by NRS/ER or RHM) were mutually exclusive, except for nine instances, largely found in the Alveolata superphylum (Fig. 4). In general, our analysis suggests that the plant-like NRS/ER or RHM pathway is more common among algal groups than the bacteria-like RmlCD pathway.

### Primary endosymbionts

For the algae derived from primary endosymbiosis (*i.e.* glaucophytes, red algae, and green algae), no orthologs of *P. parvum* RmlCD were found. Of the two glaucophyte transcriptomes examined in this study, both contained one ortholog of NRS/ER, suggesting a plant-like biosynthesis of l-Rha in this phylum. Of the green algae examined, 6 of 13 contained an NRS/ER ortholog, and 6 of the remaining 7 contained an ortholog of the trifunctional RHM. No hits were observed for one *Micromonas* sp. strain examined. The lack of *P. parvum* RmlCD orthologs and abundance of plant NRS/ER or RHM orthologs would also support a plant-like biosynthesis of l-Rha in this taxonomic group. Of the 11 red algae examined, only 3 contained orthologs of NRS/ER, with no orthologs of RmlC or RHM being observed, which may suggest a large-scale loss of l-Rha biosynthesis in this group.

### Secondary endosymbionts—green algal plastids

Among algae derived from secondary endosymbiosis with a green algal symbiont (*i.e.* excavates and rhizarians), two of three excavates examined contained NRS/ER orthologs, with no instances of RmlCD orthologs. No hits were observed for *Eutreptiella gymnastica*. This suggests a plant-like l-Rha biosynthesis pathway again. In Rhizaria, an unexpected recurring transcript was found that appears to be a fusion between RmlC and NRS/ER. Of the seven rhizarians examined, six contained this RmlC/NRS/ER putative fusion protein. In addition to this transcript, *Gymnochlora* sp. also contained a stand-alone NRS/ER ortholog. No hits for l-Rha biosynthesis were found for *Chlorarachnion reptans*. Rhizaria therefore appear to combine both bacterial and plant-like machinery for l-Rha biosynthesis.

### Secondary endosymbionts—red algal plastids

Among algae derived from secondary endosymbiosis with a red alga (*i.e.* CASH), the cryptophytes all contained NRS/ER orthologs (15/15). Of these 15, *Rhodomonas salina* also had an ortholog of *P. parvum* RmlCD. The abundance of NRS/ER orthologs and lack of RmlCD orthologs in this group would suggest a plant-like biosynthesis of l-Rha in the cryptophytes.

Of the 21 haptophytes examined, only 2 contained orthologs of either NRS/ER or RHM. Unexpectedly, 16 of the 21 examined haptophytes contained sequences orthologous to *P. parvum* RmlCD. No hits were found for l-Rha biosynthesis in *Pavlova lutheri*, *Pavlova* sp., one strain of *Chrysochromulina polyepsis*, and *Phaeocystis* sp. Taken together, this suggests a bacteria-like pathway for l-Rha biosynthesis in the haptophytes, like that seen for *P. parvum*.

The stramenopiles displayed a similar pattern to the cryptophytes, with 42 of the 47 strains examined containing an ortholog of NRS/ER. Of these 42, *Amphiprora* sp. and *Chaetoceros neogracile* also contained a RmlCD fusion ortholog. Five strains examined contained no hits to RmlCD, NRS/ER, or RHM. This consistent abundance of NRS/ER orthologs would also support a plant-like l-Rha biosynthesis pathway in the stramenopiles.

Unlike the other groups, a mix of bacterial and plant-like l-Rha biosynthetic machinery was observed for the alveolates. Of the 32 strains examined, 25 had orthologs of NRS/ER, RHM, or both, suggesting plant-like pathways are present consistently in this superphylum. However, bacterial pathways represented by *P. parvum* RmlCD also appeared, with 6 of 38 strains having an ortholog to *P. parvum* RmlCD. Interestingly, all dinoflagellates examined from the Gymnodiniaceae family (*Amphidinium*, *Karenia*, and *Karlodinium* genera) contained this RmlCD fusion; the possible origin of this fusion protein is discussed later. The dinoflagellates, which make up a subgroup of the alveolates, represent a phylum that has undergone extensive endosymbiotic events, and this may explain the abundance of both bacterial and plant-like l-Rha biosynthesis pathways in this group.

In summary, our findings suggest that most algae use exclusively the plant-like, NRS/ER or RHM pathway for l-Rha biosynthesis. One exception is the haptophytes, which appear to use a variation of the bacterial pathway. A second exception is the Rhizaria, which contain a fusion between bacterial RmlC and plant NRS/ER. Finally, some members of the Alveolata, such as the Gymnodiniaceae family, possess the plant-like pathway as well as the bacterial pathway found in the haptophytes.

### Phylogenetic analysis and evolutionary analysis

**Figure 5.**
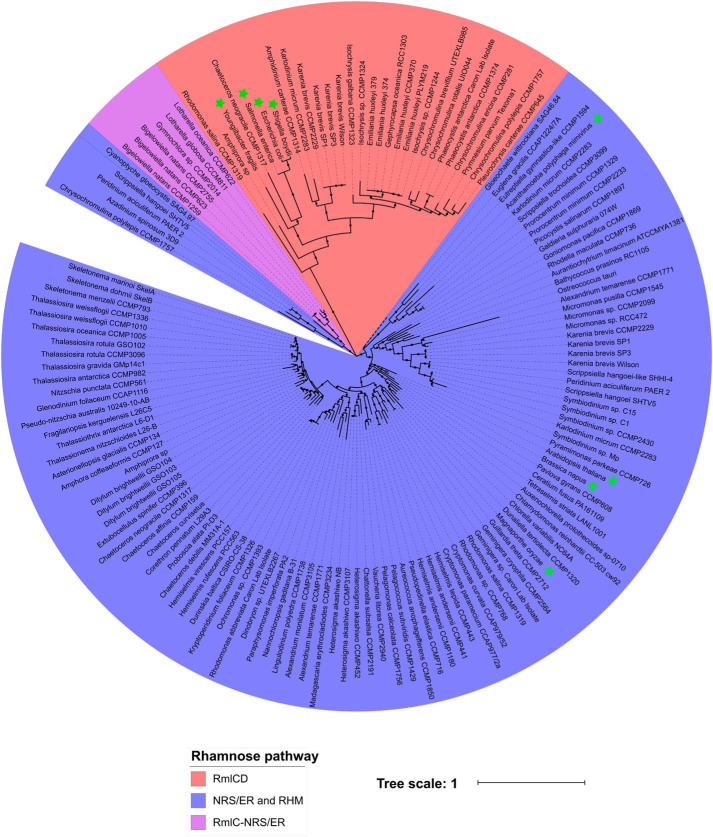
**Phylogenetic clustering of NDP-β-l-Rha biosynthetic machinery.** Protein sequences orthologous to RmlCD from *P. parvum* are highlighted in *red*, sequences orthologous to plant NRS/ER or RHM are highlighted *blue*, and sequences from the Rhizaria orthologous to an RmlC-NRS/ER fusion are highlighted *purple*. Eight sequences from plants, viruses, and bacteria used for comparison are marked with *green stars*. Alignment was performed using the default settings of MAFFT (48), and an unrooted maximum-likelihood phylogenetic tree was produced using 143 sequences from algae, bacteria, and plants. The tree was drawn using MEGA7 (49). The final tree was based on 119 ungapped amino acid positions; 100 resampling permutations and branches with bootstrap support <50% are labeled with a *black circle*. A detailed list of sequences used to create this tree can be found in Table S2.

A maximum likelihood tree of representative RmlCD, NRS/ER, and RHM orthologs was constructed using previously acquired algal sequences (referred to above), along with orthologous sequences from selected bacteria and plants. Sequences representing RmlCD chimeric proteins from bacteria and algae clearly do not form monophyletic lineages with plant-like NRS/ER or RHM sequences (Fig. 5). NRS/ER or RHM orthologs from other algal groups including the cryptophytes, alveolates (peridinin-containing dinoflagellates), stramenopiles, excavates, glaucophytes, green algae, and red algae cluster with plant sequences from *Arabidopsis thaliana*, *Brassica napus*, and *Magnaporthe oryzae*, as well as with a viral sequence from *Acanthameoba polyphaga* minivirus. Sequences from the Rhizaria predicted to be a fusion of RmlC and NRS/ER do not cluster with either the RmlCD or NRS/ER and RHM sequences. Instead, these sequences cluster with a poorly resolved (branches with bootstrap support <50%) subset of NRS/ER sequences. These results support the assignment of the *P. parvum* RmlCD-like sequences to the bacterial-like l-Rha biosynthetic pathway, and the NRS/ER and RHM sequences to the plant-like l-Rha biosynthetic pathway. The origin of the RmlC/NRS/ER fusion seen in the Rhizaria is difficult to interpret from this tree alone.

## Discussion

The biosynthesis of l-Rha has been well-described for bacteria (34), fungi (8, 35), plants (18, 19), and viruses (20). It is therefore somewhat surprising that little effort has been made to decipher l-Rha biosynthetic pathways in algae, despite the well-documented presence of l-Rha in these organisms (22, 23, 27). To fill this knowledge gap, we first sought to determine the identities of nucleoside-activated l-rhamnose in two representative algae, *E. gracilis* and *P. parvum*. To this end, we set out to quantify the intracellular levels of NDP-β-l-Rha metabolites in the two algal species.

We employed a chemical synthesis strategy to obtain the target compounds UDP-β-l-Rha and TDP-β-l-Rha, to be used as authentic standards required in metabolite profiling. We employed a known synthesis of β-l-rhamnopyranosyl phosphate (36) and this compound was used as a common precursor for the synthesis of both TDP-β-l-Rha and UDP-β-l-Rha (Scheme 1).

The LC-MS/MS method for sugar-nucleotide profiling used here takes advantage of the ability of surface-conditioned porous graphitic carbon to separate these polar metabolites using a MS-compatible buffer (30). The more hydrophobic nature of thymidine with respect to uridine led to good separation of the corresponding TDP- and UDP-sugar species. More importantly, however, separation was also achieved for NDP-l-Rha and NDP-d-glucose species bearing the same nucleobase. *E. gracilis* was found to have ∼260 times more UDP-β-l-Rha than TDP-β-l-Rha, whereas *P. parvum* was found to contain ∼6 times more TDP-β-l-Rha than UDP-β-l-Rha. Interestingly, however, both species displayed notable levels of both TDP and UDP activated l-Rha. This would support *in vitro* work on both pathways that shows reduced, but still appreciable, activities for the alternate nucleotide in each pathway (1, 18, 19). Another possibility for the difference in choice of the activating nucleobase between the two algae was the availability of the starting compound, either UDP-α-d-Glc or TDP-α-d-Glc. For this reason, we also investigated the levels of these two sugar nucleotides, but no obvious correlation was found in the levels of UDP- or TDP-α-d-Glc that may explain the differences in activated Rha levels.

Turnock and Ferguson (28) showed that *Trypanosoma cruzi* produces the UDP activated form of l-Rha, which is ∼28 times less abundant than its precursor, UDP-α-d-Glc, in this species. This value is strikingly similar to the value presented here for *E. gracilis*, also a protozoan and an evolutionary relative of the trypanosomes (26, 37), which has ∼23 times more UDP-α-d-Glc than UDP-β-l-Rha (Fig. 2). Interestingly, for *P. parvum*, TDP-β-l-Rha is ∼250 times more abundant than TDP-α-d-Glc, suggesting an as yet undefined but significant role for l-Rha in *Prymnesium* glycobiology. This prospect is somewhat at odds with the early findings of Marker (27), who claimed that l-Rha is only present in trace amounts in the extracellular preparations of *P. parvum*, with none observed inside the cell. For *E. gracilis*, early work by Barras and Stone (23), among others (24), confirmed the presence of l-Rha in the pellicle and mucus. This work was later built upon by Nakano *et al.* (25), who showed l-Rha to be the most abundant monosaccharide in the pellicle of *E. gracilis*. The lack of correlation between relative levels of activated d-Glc to l-Rha sugar nucleotides would suggest that *E. gracilis* and *P. parvum* have evolved independent routes for l-Rha biosynthesis.

To explain our metabolomics findings, we next sought to determine the presence of NDP-β-l-Rha biosynthetic machinery in *E. gracilis* and *P. parvum*, hypothesizing a plant-like NRS/ER or RHM machinery in *E. gracilis* and a bacteria-like Rml machinery in *P. parvum*. BLASTp analysis using known RmlC, NRS/ER, and RHM sequences as consensus sequences suggested that *E. gracilis* contained an ortholog to NRS/ER, whereas *P. parvum* contained a novel chimeric protein of RmlC and RmlD. To confirm the function of the new RmlCD chimera from *P. parvum*, we next sought to assess the biochemical function of this RmlCD fusion protein by heterologous expression in *E. coli* and subsequent biochemical characterization. Analysis of this protein using a combination of ^1^H NMR and MS showed that the enzyme was a bifunctional 3,5-epimerase/4-reductase, producing both UDP- and TDP-β-l-Rha from UDP- and TDP-6-deoxy-α-d-*xylo*-hexos-4-ulose, respectively. Given that appreciable levels of both UDP- and TDP-β-l-Rha were found in *P. parvum* using LC-MS and we were unable to find evidence for NRS/ER or RHM in transcriptome data for this species, we assume that *P. parvum* RmlCD is involved in both UDP- and TDP-β-l-Rha production *in vivo*.

Using the newly discovered *P. parvum* RmlCD sequence, we subsequently examined the distribution of plant-like NRS/ER and RHM enzymes, as well as bacteria-like *P. parvum* RmlCD, right across the algal groups. For this analysis 151 transcriptomes from MMETSP (38) or genomes from NCBI were used, representing a diverse range of all algal groups. RmlB and UDP-Glc 4,6-dehydratase were absent from this analysis as paralogs are found in alternative sugar-nucleotide biosynthetic pathways (13, 39). It is important to note that for this analysis, lack of transcripts could be because of lack of expression under the experimental growth conditions and does not necessarily equate to lack of gene in the genome of the organism. Equally with genomic analysis, lack of genes could be because of insufficient read depth during genome sequencing.

We identified that most algal groups utilize primarily a plant-like biosynthesis of l-Rha, with transcripts for NRS/ER and RHM identified throughout the glaucophytes, red algae, green algae, excavates, cryptophytes, alveolates, and stramenopiles. In contrast, we found that the haptophytes show very little evidence for plant-like l-Rha biosynthesis; instead they operate a bacterial-like Rml biosynthesis pathway, with transcripts for a novel fusion of RmlC and RmlD abundant throughout. This fusion protein may represent a good example of gene fusion in early eukaryotes, as discussed by Yin (40). The Rhizaria are also an exception, with an unexpected fusion between bacterial RmlC and plant NRS/ER found throughout, of which the biochemical function is currently unknown. Transcripts corresponding to the trifunctional RHM are more abundant in the Alveolata superphylum. Interestingly, like the haptophytes, the Gymnodiniaceae family of dinoflagellates all contain sequences corresponding to RmlCD chimeric enzymes. This increased genetic diversity in the Alveolata could be because of the presence of tertiary or even quaternary endosymbiosis events found in the Dinoflagellata phylum (41).

To investigate the evolutionary origin of both the plant-like NRS/ER and RHM sequences and bacteria-like *P. parvum* RmlCD sequences, a maximum likelihood phylogenetic tree was constructed (Fig. 5). The tree shows a clear divergence between RmlCD and NRS/ER or RHM pathways, with the former sequences found exclusively in the Haptophyta and Gymnodiniaceae. With the exception of the Rhizaria and a select few other sequences, this tree supports a broad distribution of NRS/ER or RHM-like sequences among other algal groups, which would support an ancient evolutionary origin of this gene. Conversely, RmlCD sequences from the Haptophyta and Gymnodiniaceae branch closely with bacterial sequences, suggesting a bacterial origin of these sequences and may indicate a case of horizontal gene transfer (HGT) in the haptophytes or Gymnodiniaceae; although we cannot discount the possibility that this gene was present in the last common eukaryotic ancestor and subsequently lost in all other groups of algae (however unlikely this may be). The tree also shows that RmlCD sequences from Gymnodiniaceae branch more closely with the haptophytes than bacteria, suggesting that one instance of HGT occurred, rather than two independent instances to the haptophytes and Gymnodiniaceae. Given that the Gymnodiniaceae plastids are known to have derived from tertiary endosymbiosis with haptophytes (42, 43), it seems likely that endosymbiotic gene transfer of RmlCD has occurred from haptophytes to this family of dinoflagellates. The additional presence of the NRS/ER or RHM biosynthetic machinery in the Gymnodiniaceae supports the previous two propositions. The absence of the plant-like machinery in the haptophytes would suggest the loss of this type of machinery occurred sometime after secondary endosymbiosis established this family (290 to 220 million years ago) (44).

**Figure 6.**
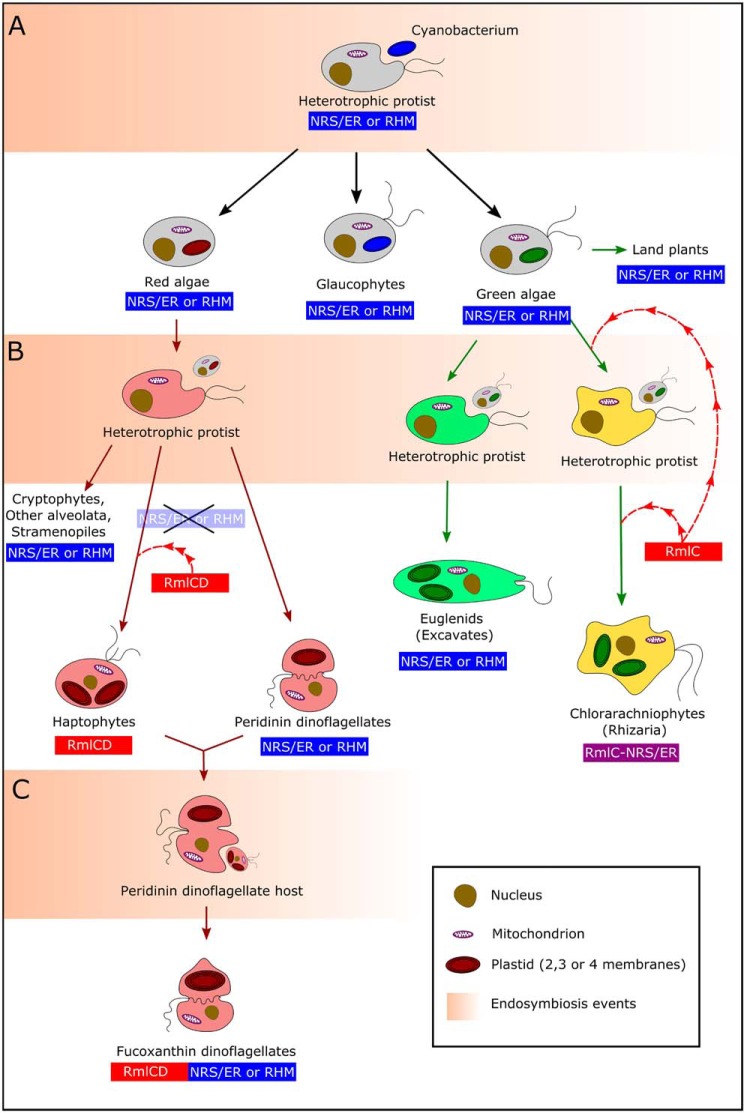
**A proposed evolutionary model of NDP-β-l-Rha biosynthesis in photosynthetic eukaryotes.** The presence of the plant-like NRS/ER or RHM machinery is denoted by a *blue box* under the group name. The presence of bacteria-like RmlC or RmlCD is denoted by a *red box* under the group name. The presence of a bacterial RmlC and plant NRS/ER fusion is denoted by a *purple box* under the group. A *dashed red arrow* indicates a likely horizontal gene transfer acquisition of a bacterial gene. *A*, primary endosymbiosis between a heterotrophic protist host and cyanobacterial symbiont, leading to emergence of red algae, glaucophytes, and green algae. *B*, secondary endosymbiosis between a heterotrophic protist host and common red algal ancestor leading to the Cryptophyta, Alveolata, Stramenopiles, and Haptophyta (CASH group of algae), two separate endosymbiotic events with different green algae leading to the Excavates and Chlorarachniophytes. *C*, example of tertiary endosymbiosis event between a peridin dinoflagellate host and a haptophyte symbiont leading to the fucoxanthin-containing dinoflagellates (Gymnodiniaceae). Nuclei are represented by a *brown circular shape*; mitochondria are represented by *purple outlined ovals*; plastids are colored according to their algal origin (*i.e. red* if derived from a red alga, *green* if derived from a green alga). The colors of the cytoplasm in algae derived from secondary or tertiary endosymbiosis are colored roughly per the scheme seen in Fig. 4.

With regard to the RmlC/NRS/ER fusion observed in the Rhizaria, the absence of RmlC in any green algae examined in this study would suggest that an independent HGT event occurred that incorporated RmlC into the genome of the Rhizaria after secondary endosymbiosis. We cannot, however, discount the possibility that the green algal symbiont had obtained this RmlC via HGT prior to secondary endosymbiosis and passed it onto the Rhizaria via endosymbiotic gene transfer. All of these evolutionary propositions are illustrated in Fig. 6.

In conclusion, in this study we show that algae, represented in this study by *E. gracilis* and *P. parvum*, contain significantly different levels of TDP- or UDP-activated l-rhamnose. This difference is likely because of different biosynthetic genes the algae contain for l-Rha biosynthesis, with *E. gracilis* containing a gene orthologous to plant-like NRS/ER and *P. parvum* containing a novel fusion of orthologs of the bacterial RmlC and RmlD enzymes. Strikingly, we provide biochemical evidence that *P. parvum* RmlCD produces both UDP- and TDP-β-l-Rha from UDP- and TDP-6-deoxy-α-d-*xylo*-hexos-4-ulose, respectively. Our comprehensive bioinformatics analysis reveals that NRS/ER and RHM sequences are widespread across algae, whereas instances of the RmlCD fusion seen in *P. parvum* are more confined to the Haptophyta and Gymnodiniaceae families. Taking these findings and the knowledge of endosymbiosis events in the algal lineages, we propose that plant-like NRS/ER or RHM sequences were likely present in a common ancestor of the algal lineages, whereas the RmlCD fusion was acquired by the Haptophytes and subsequently passed to the Gymnodiniaceae via endosymbiotic gene transfer. These results not only provide the first biochemical basis for a novel fusion protein, but also add valuable data to the currently underrepresented field of algal glycobiology.

## Experimental procedures

A full account of the synthetic approach, experimental protocols, associated analytical and spectroscopic data for the synthesis of TDP-β-l-Rha and UDP-β-l-Rha can be found in the supporting information (Figs. S2–S12). TDP-α-d-Glc, UDP-α-d-Glc, and GDP-α-d-Glc were obtained commercially from Sigma-Aldrich. UDP-α-d-GlcNAcA was synthesized as described previously (45).

### Euglena gracilis axenic cell culture

*Euglena gracilis* var. *saccharophila* Klebs (strain 1224/7a) was obtained from the Culture Collection of Algae and Protozoa (CCAP) and cultured essentially as described previously (31). Stock culture was treated with antibiotics according to a method suggested by CCAP to produce an axenic culture with small modifications to the antibiotic components (only cefotaxime, carbenicillin, and kanamycin were used). The stock culture was treated with 0, 0.5, and 1% of the antibiotic mixture in the recommended 1× *Euglena gracilis* medium (EG) + 1× Jaworski's medium (JM) for *Euglena gracilis* and subsequently inoculated into fresh 1× EG + 1× JM at the following time intervals: 24, 48, and 72 h. The culture was examined by microscopy and plating on 1× EG + 1× JM agar to confirm the production of an axenic culture.

Batch cultures (three biological replicates) were grown essentially as described before (31). In brief, cells were grown at 22 °C on a 14:10 light cycle with a light intensity of 100 μmol·m^−2^·s^−1^. Mid-log phase (*A*_600_ = 1.1 in about 6 days) cultures were harvested. Cells were pelleted by centrifugation (6750 × *g* for 20 min at 4 °C). The pellet was re-suspended in ice-cold PBS (200 ml) and centrifuged again (6750 × *g* for 20 min at 4 °C). The pellet was transferred into tared centrifuge vial (Oak Ridge) using PBS (25 ml) and centrifuged (6750 × *g* for 20 min at 4 °C), and the supernatant was carefully decanted before weighing out the wet pellet. UDP-α-d-GlcNAcA was added to the cell pellet as internal standard (1.46 nmol/g wet pellet). The cells were lysed straight away without flash freezing and/or cold storage.

### Prymnesium parvum axenic cell culture

*P. parvum* (strain 946/6) was obtained from the CCAP and maintained in the recommended f/2-Si media. Stock cultures were treated with carbenicillin (100 μg/ml) to obtain axenic cultures, which were judged to be axenic by optical microscopy. Batch cultures (three biological repeats) were grown at 22 °C on a 14:10 light cycle with a light intensity of 100 μmol·m^−2^·s^−1^, as described previously (46). Under these conditions, cell densities of ∼3 × 10^6^ cells ml^−1^ could be achieved after 12–16 days of growth. Cells were pelleted by centrifugation (6748 × *g*, 20 min, 4 °C). The pellet was transferred into tared centrifuge vial (Oak Ridge) using ice-cold PBS (20 ml), centrifuged (12,857 × *g*, 20 min, 4 °C) to give pellet. GDP-α-d-Glc was added (1.54 nmol/g wet pellet). The cells were lysed straight away without flash freezing and/or cold storage.

### Bioinformatic analysis

For the identification of NDP-β-l-Rha biosynthetic pathways, BLASTp (47) analysis was first carried out against a recently described transcriptome of *E. gracilis* (31) and a publically available transcriptome of *P. parvum* (Texoma1, MMETSP) (38). This was later expanded to include the transcriptomes (MMETSP) or genomes (NCBI) of representative algae from all algal groups (see Table S2). Protein sequences for RmlC (NP_217982.1), NRS/ER (NP_564806.1), and RHM1 (NP_177978.1) were used as consensus sequences. Hits with *E*-values ≤ 1E-10 were then manually analyzed for conserved domains before being assigned as a hit.

For phylogenetic analysis of NDP-β-l-Rha biosynthesis hits, multiple sequence alignments were generated using the default settings of MAFFT (48), including additional sequences from bacteria and plants. Regions of poor alignment were inspected for manually and their respective sequences were removed. An unrooted maximum likelihood tree was then generated using MEGA7 (49) with 100 bootstraps. The final tree was based on 119 ungapped amino acid residues and was made up of 143 sequences representing a broad diversity of the algal sequences seen in Fig. 4.

### Recombinant protein production

A vector containing UDP-glucose 4,6-dehydratase from ATCV-1 was kindly provided by Professor Michela Tonetti of the Università degli Studi di Genova, Genoa (UNIGE). The protein was expressed as a GST fusion and purified as described previously (20).

*P. parvum* RmlCD (CAMPEP_0191228776) was codon optimized for expression in *E. coli* using Integrated DNA Technologies (IDT) DNA codon optimization software (https://www.idtdna.com/CodonOpt).^4^ The resulting sequence was then synthesized with overhangs for In-Fusion® Cloning into pOPINF vector (32) using IDT's gBlock gene fragment synthesis service. The sequence used for protein expression in this study can be found in the supporting material.

The gBlock gene fragment was then cloned into pOPINF using an In-Fusion® Cloning Kit according to the manufacturer's instructions. The resulting plasmid was then transformed into Stellar competent cells before being propagated and extracted using a miniprep kit (Qiagen, Manchester, UK). Positively transformed plasmids were identified by size comparison to a nontransformed pOPINF control plasmid using agarose gel electrophoresis. A plasmid containing the gBlock sequence was then transformed into SoluBL21^TM^ competent cells (Genlantis) for protein expression. Two liters of *E. coli* cells were grown to an *A*_600_ of ∼0.5 at 37 °C before being transferred to 18 °C for 1 h. Induction was performed using 0.5 mm IPTG and cells were left at 18 °C overnight. Proteins were extracted in a buffer containing 50 mm Tris-HCl, pH 7.5, 0.5 m NaCl, 20 mm imidazole, protease inhibitor mixture (Sigma) 1/100 v/v, 2 mg DNase. *P. parvum* RmlCD was purified using nickel affinity chromatography, and fractions judged to be >95% pure by SDS-PAGE were pooled for subsequent biochemical analysis.

### Biochemical analyses

To assess the relative activity of *P. parvum* RmlCD in producing UDP- and TDP-β-l-Rha, we first needed to produce the predicted substrates, UDP- and TDP-6-deoxy-α-d-*xylo*-hexos-4-ulose. Reactions contained 4 mm UDP/TDP-α-d-glucose, 6.4 mm NADPH (RmlCD cofactor), 6.4 mm NAD^+^, 60 μg ATCV-1 UGD/200 μg *S. enterica* Typhimurium RmlB and 2 mm MgCl_2_ buffered in 50 mm HEPES, pD 7.5. Addition of NAD^+^ was not required for RmlB activity but was included in both reactions for completeness. Reactions were monitored by ^1^H NMR until the anomeric signal for UDP- or TDP-α-d-glucose (5.55 ppm) had disappeared, at which point 40 μg of RmlCD was added to the reaction mixtures. The reactions were monitored by ^1^H NMR to monitor loss of anomeric signals representing UDP- or TDP-6-deoxy-α-d-*xylo*-hexos-4-ulose (keto, 5.68 ppm; hydrate, 5.48 ppm) and formation of new anomeric signals from UDP- or TDP-β-l-Rha (5.16 ppm). Relative rates of formation of UDP- or TDP-β-l-Rha by RmlCD were calculated by signal integration of the anomeric protons of UDP- or TDP-6-deoxy-α-d-*xylo*-hexos-4-ulose to the anomeric protons of UDP- or TDP-β-l-Rha. MS of the TDP-β-l-Rha reaction in a 10% D_2_O buffer revealed the expected mass for TDP-β-l-Rha (HRMS, ESI negative: calculated: 547.0736, found: 547.0737, 0.2 ppm). Note: when reactions were repeated in 80% D_2_O-based buffers, mass values increased by +1, +2, +3, or +4 corresponding to incorporation of deuterium onto the sugar ring system as reported previously (50). TDP-β-l-Rha was purified using strong anion exchange as outlined in Wagstaff *et al.* (51). ^1^H NMR values agreed well with our synthetic TDP-β-l-Rha values (Figs. S9 and S10) and also published literature values (52, 53).

### Sugar-nucleotide extraction and profiling

A full account of the methodology for sugar-nucleotide profiling can be found in the supporting material. Sugar nucleotides were extracted and profiled essentially as described previously (54). Axenic cultures of algae were grown under standardized conditions. The cells were harvested, and a known amount of internal standard was added. Intracellular sugar nucleotides were extracted using lysis with cold 70% EtOH followed by de-fatting and solid phase extraction. LC-ESI-MS/MS was used to analyze and quantify the intracellular sugar nucleotides. Separation was achieved on a surface-conditioned porous graphitic carbon column (Hypercarb) and the target analytes were detected by negative electrospray ionization using Xevo TQ-S tandem quadrupole mass spectrometer operated in MRM mode. Authentic standards of sugar nucleotides were used to generate MRM transitions and to determine retention times. A detailed description of the sugar nucleotides extraction and profiling can be found in the supporting information.

## Author contributions

B. A. W., M. R., and R. A. F. conceptualization; B. A. W., M. R., S. K., L. H., and I. M. data curation; B. A. W., M. R., S. A. N., H. C. D., and R. A. F. formal analysis; B. A. W. and R. A. F. validation; B. A. W. and M. R. investigation; B. A. W. visualization; B. A. W., M. R., S. K., L. H., and S. A. N. methodology; B. A. W. writing-original draft; B. A. W., M. R., S. K., H. C. D., and R. A. F. writing-review and editing; M. R., S. A. N., H. C. D., and R. A. F. supervision; H. C. D. and R. A. F. resources; H. C. D. and R. A. F. funding acquisition; H. C. D. and R. A. F. project administration.

## Supplementary Material

Supporting Information

## Footnotes

4 Please note that the JBC is not responsible for the long-term archiving and maintenance of this site or any other third party hosted site.

## Footnotes

3 The abbreviations used are:

Rha: rhamnose
MRM: multiple reaction monitoring
ESI: electrospray ionization
NRS/ER: nucleotide–rhamnose synthase/epimerase-reductase
HGT: horizontal gene transfer
CCAP: Culture Collection of Algae and Protozoa
EG: *Euglena gracilis* medium
JM: Jaworski's medium
MMETSP: Marine Microbial Eukaryote Transcriptome Sequencing Project.
