# Supplementary material for: Discovery of an RmlC/D fusion protein in the microalga Prymnesium parvum and its implications for NDP-β-l-rhamnose biosynthesis in microalgae
Source: J Biol Chem. 2019 Apr 22;294(23):9172–85. doi: 10.1074/jbc.RA118.006440 (PMC6556577; doi:10.1074/jbc.RA118.006440)
Supplement: Supporting Information [file supp_294_23_9172__index.html]

Discovery of an RmlC/D fusion protein in the microalga Prymnesium parvum and its implications for NDP-β-L-rhamnose biosynthesis in microalgae — NDP-β-L-rhamnose biosynthesis in algae — Discovery of an RmlC/D fusion protein in the microalga Prymnesium parvum and its implications for NDP-β-l-rhamnose biosynthesis in microalgae — NDP-β-l-rhamnose biosynthesis in algae — Supporting Information 

# Discovery of an RmlC/D fusion protein in the microalga *Prymnesium parvum* and its implications for NDP-β-l-rhamnose biosynthesis in microalgae

## Supporting Information

- Supporting Information (to be published online) - Supporting Information
