# Supplementary material for: Discovery of an RmlC/D fusion protein in the microalga Prymnesium parvum and its implications for NDP-β-l-rhamnose biosynthesis in microalgae
Source: J Biol Chem. 2019 Apr 22;294(23):9172–85. doi: 10.1074/jbc.RA118.006440 (PMC6556577; doi:10.1074/jbc.RA118.006440)
Supplement: Supporting Information [file supp_RA118.006440_141574_2_supp_317518_pq7cg2.pdf]

Supplementary material

Article title: **Discovery of an RmlC/D fusion protein in the microalga *Prymnesium parvum* and its implications for NDP-β-L-rhamnose biosynthesis in microalgae**

Authors: Ben A. Wagstaff[1,2‡], Martin Rejzek[1‡], Sakonwan Kuhaudomlarp[1,3], Lionel Hill[1], Ilaria Mascia[1], Sergey A. Nepogodiev[1], Helge C. Dorfmueller[2] and Robert A. Field[1] *

From the [1]Department of Biological Chemistry, John Innes Centre, Norwich Research Park, Norwich, NR4 7UH, United Kingdom; [2]Division of Molecular Microbiology, School of Life Sciences, University of Dundee, Dundee, DD1 5EH, United Kingdom; [3]Université Grenoble Alpes, CNRS, CERMAV, 38000, Grenoble, France

**To whom correspondence should be addressed: Robert A. Field: Department of Biological Chemistry, John Innes Centre, Norwich Research Park, Norwich, NR4 7UH, UK; rob.field@jic.ac.uk; tel. (+44)-1603-450720*

‡ These authors contributed equally to this study.

Article acceptance date: XXX

List of contents of Supplementary Material

Supplementary material 1
List of contents of Supplementary Material 2
Introduction 4
Results and Discussion 7
Experimental 10
General Methods 10
Sequence used for protein expression 11
Chemical synthesis 13
Sugar nucleotide extraction and profiling 16
References 41

Figure S1 – The target nucleotide-diphospho-sugars and their numbering……………4

Figure S2 - SDS-PAGE analysis of Nickel column purified *P. parvum* RmlCD produced in *E. coli* in this study ……………………………………………………………………………19

Figure S3 - Production of UDP-β-L-Rha using ATCV-1 UDP-glucose 4,6-dehydratase and *P. parvum* RmlCD ………………………………………………………………………….…19

Figure S4 - ^{1}H NMR spectrum ($CDCl_3$, 400 MHz) of 2,3,4-tri-*O*-acetyl-L-rhamnopyranose (4)…………………………………………………………………………………….....20

Figure S5 - ^{1}H coupled ^{13}C NMR of 5β and 5α in toluene-d8…………………………..….21

Figure S6 - NOESY spectrum of 5β in toluene-d8………………………………………21

Figure S7 - NOESY spectrum of 5α in toluene-d8………………………………………22

Figure S8 - Stacked ^{1}H NMR spectra of 5α and 5β in toluene-d8………………….…..….22

Figure S9 - ^{1}H NMR spectrum (D_2O, 400 MHz) of TDP-β-L-Rhamnose bistriethylammonium salt (1)...23

Figure S10 - ^{1}H NMR spectrum (D_2O, 400 MHz) of TDP-β-L-Rhamnose produced enzymatically in this study…………………………………………………………………..23

Figure S11 - ^{1}H NMR spectrum (D_2O, 400 MHz, internal acetone at δ_H 2.22 ppm) of UDP-β-L-Rhamnose bistriethylammonium salt (2)..24

Figure S12 - ^{13}C NMR spectrum (D_2O, 100 MHz, internal acetone at δ_C 30.89 ppm) of UDP-β-L-Rhamnose bistriethylammonium salt (2). 24

Table S1 – Relative retention times and MRM transitions of sugar nucleotides examined in this study. 18

Table S2 – List of organisms examined in this study with respective nucleic acid database identifiers and sequence identifiers found for NDP-β-L-Rha biosynthesis. 40

Introduction

During our studies on algal glycosylation machinery we had a need to prepare multi milligram quantities of pure thymidine 5'-diphospho-β-L-rhamnose (TDP-β-L-Rha) (**1**) and uridine 5'-diphospho-β-L-rhamnose (UDP-β-L-Rha) (**2**) (Figure S1).

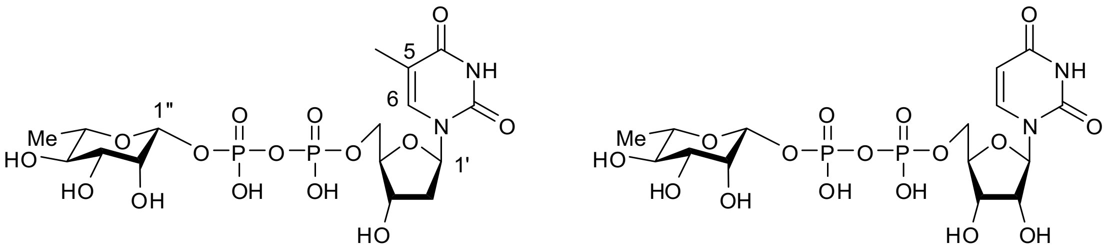

TDP-β-L-Rha
1

UDP-β-L-Rha
2

Figure S1– The target nucleotide-diphospho-sugars and their numbering.

From a synthetic point of view the common feature of the target nucleotide-diphospho-sugars **1** and **2** is the 1,2-cis configuration of the sugar ring that is difficult to make and once installed the resulting compounds are very acid labile with a high tendency to hydrolyse or to form cyclic 1,2-phosphodiesters. The key to the synthesis of compounds **1** and **2** is the preparation of the corresponding sugar-1-phosphate **6** (Scheme 1) with well-defined stereochemistry of the anomeric centre.

Currently available approaches to TDP-β-L-Rha (**1**) employ enzymatic transformation of TDP-α-D-Glc and the product of this transformation has been isolated and characterised reasonably well by ^{1}H NMR [1-3].

A chemical synthesis of TDP-β-L-Rha (**1**) has been published by Shibaev *et al* [4] using a coupling of β-L-rhamnopyranosyl phosphate [5] with either TMP-morpholidate or thymidine 5'-diphenylpyrophosphate. The same protocol was employed to prepare UDP-β-L-Rha (**2**). Unfortunately, apart from mobility in paper chromatography, paper electrophoresis and nucleoside / total phosphate / acid-labile phosphate ratios the products were not further characterised. The β-L-rhamnopyranosyl phosphate used by Shibaev *et al* [4] was prepared by a slightly modified procedure [5] published earlier by Prihar & Behrman [6] that employs phosphorylation of the hemiacetal hydroxyl group in 2,3,4-tri-*O*-acetyl-β-L-rhamnopyranose by *o*-phenylene phosphorochloridate in the presence of an organic base followed by oxidation and deprotection steps. This method gives reliably the β-L-rhamnopyranosyl phosphate (although small amounts of the α-anomer are probably formed as well and need to be removed

by chromatography, (see Offen *et al* [7]) and consequently, it would be fair to assume that the resulting nucleotide-diphospho-sugars prepared by Shibaev *et al* [4] have the correct anomeric configuration. However, in the absence of the analytical data to support this, uncertainty remains. A more recent synthesis of TDP-β-L-Rha (**1**) was published by Zhao and Thorson [8] starting from L-rhamnopyranosyl phosphate that in turn was prepared from 2,3,4-tri-*O*-acetyl-α-L-rhamnopyranosyl bromide by a silver triflate promoted phosphorylation with dibenzyl phosphate in the presence of 2,4,6-collidine followed by a global deprotection. Given a certain ambiguity in the anomeric assignment of the sugar-1-phosphate generated by Zhao and Thorson [8] later Timmons and Jakeman [9] repeated this phosphorylation using exactly the same conditions. Based on ^{1}H and ^{13}C NMR data they isolated the same product as Zhao and Thorson [8] but assigned the product an α-configuration as suggested by a one bond ^{13}C-^{1}H coupling constant ($^{1}J_{C1,H1}$ = 170 Hz) and 1D NOE. This is further supported by the fact that, in our hands, a deprotonation of the hemiacetal hydroxyl group in 2,3,4-tri-*O*-acetyl-L-rhamnopyranose (**4**) (Scheme 1) using lithium diisopropyl amide and phosphorylation of the resulting salt with tetrabenzyl pyrophosphate at -80 °C in THF gave a single anomer of 2,3,4-tri-*O*-acetyl-L-rhamnopyranosyl phosphate which by TLC, optical rotation and ^{1}H, ^{13}C and ^{31}P NMR was the same compound assigned by Zhao and Thorson [8] as the β-anomer. The one bond C1-H1 coupling constant $^{1}J_{C1,H1}$ = 178.5 Hz, however, clearly indicated the α-anomer.

As already suggested by Wagner *et al* [10] the resulting nucleotide-diphospho-sugar prepared by Zhao and Thorson [8] but also by Meyers and Borch [11] is in fact the unnatural TDP-α-L-Rha. This becomes immediately apparent when ^{1}H NMR spectra of the product made by Zhao and Thorson [8] is compared to the material generated by enzymatic reactions [1-3]. In summary all synthetic approches to TDP-β-L-Rha (**1**) published to date are in fact either producing the unnatural anomer TDP-α-L-Rha or are at least ambiguous as to the configuration of the anomeric centre. Consequently, there is a need for an alternative synthetic route to TDP-β-L-Rha (**1**).

UDP-β-L-Rha (**2**) has been generated by enzymatic conversion of UDP-α-D-Glc on numerous occasions and characterised well by NMR spectroscopy (for example most recently by Martinez *et al* [12]). The one bond C1''-H1'' coupling constant $^{1}J_{C1'',H1''}$ was determined to be 161 Hz by coupled ^{13}C-^{1}H HSQC [12].

A chemical synthesis of UDP-β-L-Rha (**2**) was published by Barber and Behrman [13] employing a condensation of uridine 5'-diphenylpyrophosphate and β-L-rhamnopyranosyl phosphate. β-L-Rhamnopyranosyl phosphate was prepared by phosphitylation of the hemiacetal hydroxyl group in 2,3,4-tri-*O*-acetyl-β-L-rhamnopyranose by 2-cyanoethyl-*N*,*N*-diisopropylchlorophosphoroimidate followed by an oxidation using *tert*-butylhydroperoxide

and LiOH hydrolysis of the acetates. Offen *et al* [7] have prepared UDP-α,β-L-Rha by phosphorylation of 2,3,4-tri-*O*-acetyl-L-rhamnopyranose with *o*-phenylene phosphorochloridate, deprotection and elaboration to the required nucleotide-diphospho-sugar.

Here, we wish to report the first fully synthetic approach to TDP-β-L-Rha (**1**) that was also modified for the preparation of UDP-β-L-Rha (**2**).

Results and Discussion

Diphenyl (tri-*O*-acetyl-β-L-rhamnopyranosyl) phosphate [14] (**5**) (Scheme 1) was used as the starting point for our syntheses of both TDP-β-L-Rha (**1**) and UDP-β-L-Rha (**2**). Because of the above-mentioned issues with previous reports regarding the anomeric configuration of the synthetic TDP-L-rhamnose we decided to prepare and purify both anomers of **5** and confirm the anomeric configuration using extensive NMR studies.

L-Rhamnose was first subjected to a one-pot procedure comprising an acetylation, followed by bromination and hydrolysis of the corresponding tri-*O*-acetyl-L-rhamnosyl bromide to yield tri-*O*-acetyl-L-rhamnopyranose (**4**) which was obtained as an anomeric mixture containing 85% of the thermodynamically more stable α anomer (α/β anomers 5.7:1 by ^{1}H NMR).

To prepare the β phosphate **5β** Sabesan and Neira used the fact that the β anomer of hemiacetal **4** is more reactive than the dominant α anomer and performed the subsequent phosphorylation at room temperature and limiting amount of the phosphorylating agent, diphenyl chlorophosphite [14]. In our hands these conditions resulted in an anomeric mixture of the diphenyl (tri-*O*-acetyl-α,β-L-rhamnopyranosyl) phosphates (**5**) that was indeed significantly enriched in the desired β anomer (α/β anomers 1:4 by ^{1}H NMR). Moreover, both anomers separated very well on silica gel TLC (**5α** R_f = 0.65 and **5β** R_f = 0.58, see Experimental section for details) and were reasonably stable provided that the mobile phase contained a small amount of Et_3N (1%) to account for limited acid stability of the phosphates. Preparative silica gel column afforded α–anomer **5α** (6%) and β–anomer **5β** (49%) in pure forms. Analytical data ($[\alpha]_D$ in $CHCl_3$, ^{1}H and ^{13}C NMR in $CDCl_3$) of both anomers were in good agreement with data published by Sabesan and Neira [14]. We have noticed, however, that both anomers of **5** undergo gradual decomposition on long exposure to chloroform or when solvent evaporation was attempted which we put down to traces of HCl present in this solvent degrading the acid labile phosphates. For this reason we acquired a second set of analytical data using toluene or toluene-d8, respectively. The anomeric phosphates **5** were stable in this solvent.

Regarding the anomeric configuration of the phosphate derivatives **5α** and **5β**, the vicinal coupling constants between H1 and H2, and vicinal coupling between H1 and P were only marginally different in ^{1}H NMR spectra of **5α** ($^3J_{1,2}$ = 1.3 Hz, $^3J_{1,P}$ = 6.7 Hz) and **5β** ($^3J_{1,2}$ = 1.2 Hz, $^3J_{1,P}$ = 6.9 Hz) and couldn't therefore be used to determine the configuration of the anomeric centre. Following Sabesan and Neira, chemical shifts of H3 and H5 in ^{1}H NMR spectra were used as reporter groups for this assignment [14]. The 1,3-*syn* diaxial orientation of H3 and H5 to the diphenylphosphate group in **5α** (H3 5.56-5.52 ppm and H5 4.01 ppm)

resulted in significant deshielding as compared to the 1,3-*anti* diaxial orientation in **5β** (H3 5.02 ppm and H5 3.13 ppm) (Figure S8). We further supported the assignment of the anomeric phosphates **5α** and **5β** by a significantly different one bond C1-H1 coupling constant in ^{1}H coupled ^{13}C NMR spectra. In agreement with literature [15-17] this coupling was smaller for **5β** ($^{1}J_{C1,H1}$ = 165.7 Hz) when compared to **5α** ($^{1}J_{C1,H1}$ = 179.4 Hz) (Figure S5). Observation of NOE contacts H1–H3 and H1–H5 in NOESY spectrum of **5β** further supported the β-anomeric configuration (Figure S6). In **5α** no NOE contact for H1 was detectable (Figure S7).

The phosphate **5β** was globally deprotected using the same conditions as in Sabesan and Neira [14] to give the desired β-L-rhamnopyranosyl phosphate (**6**). The PtO_2 catalysed hydrogenation to deprotect the diphenyl phosphate in **5β** was fast and complete even at low hydrogen pressure (around 15 psi, originally used 55 psi). In summary, the presented synthesis of β-L-rhamnopyranosyl phosphate (**6**) offers a short and a much simpler alternative to methods published earlier [6, 13]. Although perhaps not the most obvious choice, the phenyl protecting groups in the phosphates **5α/5β** greatly enhance the separation of the anomers on silica gel when compared to benzyl analogues and do not present any hurdles in terms of removal. Moreover, diphenyl chlorophosphate was commercially available whereas dibenzyl chlorophosphate was not at the time of manuscript preparation. The presented preparation of β-L-rhamnopyranosyl phosphate (**6**) is scalable to multi-gram quantities and the product in the bistriethylammonium form is stable to store over long periods of time.

The final step in the synthesis involved the standard pyrophosphate bond formation [18] between the pyridine soluble bistriethylammonium salt of β-L-rhamnopyranosyl phosphate (**6**) and the morpholidate activated [19] thymidine or uridine 5'-monophosphate, respectively, in dry pyridine. After 5 to 7 days stirring at 4 °C the yields (by HPLC) of the products were 55% for **1** and 70% for **2**. An attempted purification of the desired nucleotide-diphospho-sugars **1** and **2** using strong anion exchange chromatography failed because of considerable degree of decomposition during the freeze-drying and vacuum assisted removal of the volatile ammonium bicarbonate buffer. Instead, a combination of Sephadex LH-20 gel permeation chromatography followed by C18 reverse phase chromatography was required to achieve a complete separation of the target compounds **1** and **2** from particularly the unreacted starting materials. After purification the resulting TDP-β-L-Rha (**1**) and UDP-β-L-Rha (**2**) were obtained in yields 16% for **1** and 21% **2** in high degree of purity as shown by NMR (Figure S9, S11, S12) and HPLC (data not shown). ^{1}H NMR of TDP-β-L-Rha (**1**) produced enzymatically in this study (Figure S10) is in good agreement with synthetically produce TDP-β-L-Rha (**1**) (Figure S9).

The 1*H*-tetrazole catalysed variant [20] of the pyrophosphate coupling has also been investigated. Although shorter reaction times were required to achieve similar conversions, the products were more difficult to purify from the reaction mixtures as they were more complex than the reaction mixtures obtained from the non-catalysed variant.

Experimental

Uridine 5'-monophosphomorpholidate 4-morpholine-*N*,*N'*-dicyclohexylcarboxamidine salt (UMP-morpholidate) was purchased from Sigma-Aldrich. Thymidine 5'-monophosphomorpholidate 4-morpholine-*N*,*N'*-dicyclohexylcarboxamidine salt (TMP-morpholidate) was prepared following a published procedure [19].

TLC was performed on pre-coated silica plates (Merck 60 F254, 0.25 mm) containing a fluorescence indicator. Compounds were visualized by UV 254 nm and by dipping in H_2SO_4 (5% v/v) solution in EtOH and heating. Column chromatography on silica gel was performed using Biotage SP4 system and Biotage SNAP flash cartridges.

Optical rotations were measured at ambient temperature on a Perkin-Elmer model 341 polarimeter using a sodium lamp.

NMR spectra were recorded on a Bruker Avance III 400 MHz spectrometer. Chemical shifts of ^{1}H NMR signals recorded in D_2O are reported with respect to residual HDO at δ_H 4.70 ppm, if not stated otherwise. Chemical shifts of ^{1}H NMR signals recorded in toluene-d8 are reported with respect to the CH_3 resonance δ_H 2.11 ppm of the residual solvent. Chemical shifts of ^{13}C NMR signals recorded in D_2O are reported with respect to the methyl carbon of internal acetone at δ_C 30.89 ppm. Chemical shifts of ^{13}C NMR signals recorded in toluene-d8 are reported with respect to the CH_3 resonance δ_C 21.10 ppm of the residual solvent. Assignments were made with the aid of COSY and HSQCed experiments. In ^{13}C NMR spectra the information on multiplicity of carbon atom substitution with hydrogens (s = C, d = CH, t = CH_2, q = CH_3) has been derived from HSQCed experiments. Chemical shifts of ^{31}P NMR signals recorded in D_2O are reported with respect to external 85% H_3PO_4 at δ_P 0 ppm.

High resolution accurate mass spectra were obtained using a Synapt G2 Q-Tof mass spectrometer using either positive or negative electrospray ionisation.

General Methods

General Method A: Strong anion-exchange (SAX) HPLC on Poros HQ 50

The chromatography was performed on a Dionex Ultimate 3000 instrument equipped with UV/vis detector. An aqueous solution of a sample was applied on a Poros HQ 50 column (50×10 mm, column volume (CV) = 3.9 ml). The column was first equilibrated with 5 CV of 5 mM ammonium bicarbonate buffer, followed by linear gradient of ammonium bicarbonate from 5 mM to 250 mM in 15 CV, then hold for 5 CV, and finally back to 5 mM ammonium bicarbonate in 3 CV at a flow rate of 8 ml/min and an online UV detection to monitor A_{265}. After multiple

injections, the column was washed with 3 CV of 1 M ammonium bicarbonate followed by 3 CV of Milli-Q water.

General Method B: Gel filtration chromatography on Sephadex LH-20

The purification was performed on a Perkin Elmer Series 200 instrument equipped with UV/vis and RI detectors. A solution of a sample in MeOH was applied on a Sephadex LH-20 column (800×16 mm) and eluted isocratically with MeOH at a flow rate of 1 ml/min and detection with on-line RI detector and UV detector to monitor A_{265}. Fractions containing the sugar nucleotide (the sugar nucleotides eluted typically around 130 ml) were pooled and the solvent was evaporated *in vacuo*.

General Method C: Reverse phase (RP) C18 purification

The purification was performed on a Dionex Ultimate 3000 instrument equipped with UV/vis detector. A solution of a sample in water was applied on a Phenomenex Luna 5 µm C18(2) column (250x10 mm, CV = 19.6 ml) and eluted isocratically with 50 mM Et_3NHOAc, pH 6.8 with 1.5% CH_3CN in 8 CV at a flow rate of 5 ml/min and detection with on-line UV detector to monitor A_{265}. Fractions containing the sugar nucleotide were pooled and freeze-dried.

Sequence used for protein expression

Codon optimized *P. parvum* RmlC/D (CAMPEP_0191228776) – E.coli

ATGGCTCCTGCAACAACCGATCTGGCTGTTGATGGAGCGTCTGTAACTACTTTTGACCG
TTTTGGAGATTCTCGTGGGTACTTCAATGAGCTGTATAATGAAACTAAATACGACGGATC
GCTGGTAAAGGAGTGGAAGCAGGTATCGTTCAGTTCTTCCGGCAAGCATACTTTGCGT
GGGTTACACTGCAGCCCTTACGGGAAATTTATCACATGCGTCCGCGGGGCGTTCTATG
ATGTTATCGCTGACTTCCGCGAAGACTCTCCTACGTTCGGCCGTTGGTGTGGTGTCCTT
TTGACTGAGCATAATAAGAAACAGGTGTATGTGCCAGCTCGCTGCGGCCATGGTTTTTT
CACATTCGAAGACAATACTTGTGCTCTGTACTTACAGGAAGGGACATTCAATCCACCCG
GAGAGATGGATACCAGTCCCTTCGACTCCTTCGTAAGTGTTAAGTGGCCAATCCCCCCT
GGCGTGCAGCCTACGCTGTCTGCGAAGGATACGGTCGCCCCTCATCTGTCGGTCCGT
CGCCCCCATCTGTGTAACGCTACACCGCGTGGGCGCATTCTTATTATCGGAGCCTCAG
GGCAGGTCGGAGCAGCGTTAGTGGAAGCGTACGGGCCCCGTAATTGCATCGGCACAT
ATTCTTCAACCGCACAACCGAACATGGTACCTTTCGACTTGTCCGCAGCTGCAATGAAT
CCGCAGTTAGCAGAGGATTTGATTCAGATGGTCTACCCCACAGTAGTCTGTATCTGCGC
GGGAATGACCTGGGTAGACGGTTGCGAGCGCGACGCAGCCTTAGCAAATCGCTTGAAT
TGCGTAGGCCCGGCGGTTGTAGCTGCTGCTGCCCGCAAATTCGGCGCGAAAACGGTG
TGGTATTCCACTGATTACGTCTTCGATGGTGGCCGTAAACTTAAAAAGGGCCCTTACAC

AGAAGCGGACCCTACGAGTCCGTTGAATGTGTATGGAAGTTCAAAGTTGGAGGGTGAG
AAACAGGTTATGGCCGCAGACCCGGAAGCTCTGGTGATCCGCACGAACGTCGTTTTTG
GCCCAGAGCAGGTAGGCAAGAATTTTGTCTACCAACTTGTCCGTAAATTGAAGGCATCG
GAGAGTATGAATGTCCCCTCCGACCAGAAAAACACGCCTACCTATAACCGTGATTTAGC
CGACGCTACGAAACAGTTAGTGGAAGCTGGCGCTGTTGGGGTATTCAATGTGGGAGGT
CCGGAAGTGTTGGGTCGCGTCGAATTCGCAGAAGTAGTTGCGGATTGTCTTGGCCTGG
ACAAAAAGCCAATCGTACCGCTTACTACGGAAAATAACGGGCAAGCCGCGTTACGCCC
GCTGGACTCCGGCCTTAGTCTGGAAAAAGTTCAGGCAATTATTCCCGGCTGGCAACCT
CGTAGCGTCCGTGATGCCCTTGCGCACTGGATCGCCAACCCACGCGGGAAATTATTAG
GGGCCTAA

Modified P. parvum RmlC/D (CAMPEP_0191228776) with pOPINF cloning overhangs and no start/stop codon

AAGTTCTGTTTCAGGGCCCGGCTCCTGCAACAACCGATCTGGCTGTTGATGGAGCGTC
TGTAACTACTTTTGACCGTTTTGGAGATTCTCGTGGGTACTTCAATGAGCTGTATAATGA
AACTAAATACGACGGATCGCTGGTAAAGGAGTGGAAGCAGGTATCGTTCAGTTCTTCC
GGCAAGCATACTTTGCGTGGGTTACACTGCAGCCCTTACGGGAAATTTATCACATGCGT
CCGCGGGGCGTTCTATGATGTTATCGCTGACTTCCGCGAAGACTCTCCTACGTTCGGC
CGTTGGTGTGGTGTCCTTTTGACTGAGCATAATAAGAAACAGGTGTATGTGCCAGCTCG
CTGCGGCCATGGTTTTTTCACATTCGAAGACAATACTTGTGCTCTGTACTTACAGGAAG
GGACATTCAATCCACCCGGAGAGATGGATACCAGTCCCTTCGACTCCTTCGTAAGTGTT
AAGTGGCCAATCCCCCCTGGCGTGCAGCCTACGCTGTCTGCGAAGGATACGGTCGCC
CCTCATCTGTCGGTCCGTCGCCCCCATCTGTGTAACGCTACACCGCGTGGGCGCATTC
TTATTATCGGAGCCTCAGGGCAGGTCGGAGCAGCGTTAGTGGAAGCGTACGGGCCCC
GTAATTGCATCGGCACATATTCTTCAACCGCACAACCGAACATGGTACCTTTCGACTTG
TCCGCAGCTGCAATGAATCCGCAGTTAGCAGAGGATTTGATTCAGATGGTCTACCCCAC
AGTAGTCTGTATCTGCGCGGGAATGACCTGGGTAGACGGTTGCGAGCGCGACGCAGC
CTTAGCAAATCGCTTGAATTGCGTAGGCCCGGCGGTTGTAGCTGCTGCTGCCCGCAAA
TTCGGCGCGAAAACGGTGTGGTATTCCACTGATTACGTCTTCGATGGTGGCCGTAAACT
TAAAAAGGGCCCTTACACAGAAGCGGACCCTACGAGTCCGTTGAATGTGTATGGAAGT
TCAAAGTTGGAGGGTGAGAAACAGGTTATGGCCGCAGACCCGGAAGCTCTGGTGATCC
GCACGAACGTCGTTTTTGGCCCAGAGCAGGTAGGCAAGAATTTTGTCTACCAACTTGTC
CGTAAATTGAAGGCATCGGAGAGTATGAATGTCCCCTCCGACCAGAAAAACACGCCTA
CCTATAACCGTGATTTAGCCGACGCTACGAAACAGTTAGTGGAAGCTGGCGCTGTTGG
GGTATTCAATGTGGGAGGTCCGGAAGTGTTGGGTCGCGTCGAATTCGCAGAAGTAGTT
GCGGATTGTCTTGGCCTGGACAAAAAGCCAATCGTACCGCTTACTACGGAAAATAACG

GGCAAGCCGCGTTACGCCCGCTGGACTCCGGCCTTAGTCTGGAAAAAGTTCAGGCAAT
TATTCCCGGCTGGCAACCTCGTAGCGTCCGTGATGCCCTTGCGCACTGGATCGCCAAC
CCACGCGGGAAATTATTAGGGGCCTAAAGCTTTCTAGACCAT

Chemical synthesis

2,3,4-Tri-*O*-acetyl-L-rhamnopyranose (4)

To an ice bath (4 °C) cooled solution of L-rhamnose (**3**) (8.5 g, 51.8 mmol) in acetic anhydride (27 ml, 285.6 mmol) was added 33 wt% solution of hydrogen bromide in glacial acetic acid (15.4 ml, 89.2 mmol) slowly enough to keep the reaction temperature below 20 °C. When addition was complete the ice bath was replaced by a water bath and the mixture was stirred for 2.5 hours at 20 °C. Sodium acetate (7.4 g, 90.0 mmol) was added to neutralise excess HBr and the mixture was poured into ice-water and stirred for 1 hour. The crude product was extracted with dichloromethane (3 x 20 ml) and the combined extracts were washed with saturated aqueous $NaHCO_3$ solution. The organic layer was dried over Na_2SO_4 and the solvent was evaporated to give an amorphous solid. Crystallisation from diethyl ether gave the title compound **4** (4.4 g, 29.3%). ^{1}H NMR ($CDCl_3$, 400 MHz): δ_H 5.38 (1H, dd, $^3J_{3,4}$ = 10.0 Hz, $^3J_{3,2}$ = 3.5 Hz, H3), 5.28 (1H, dd, $^3J_{2,1}$ = 1.8 Hz, H2), 5.17 (1H, dd, $^3J_{1,OH}$ = 3.9 Hz, H1), 5.09 (1H, dd, $^3J_{4,5}$ = 10.0 Hz, H4), 4.17-4.10 (1H, m, H5), 3.15 (1H, d, OH), 2.16 (3H, s, OAc), 2.06 (3H, s, OAc), 2.00 (3H, s, OAc), 1.23 (3H, d, $^3J_{CH3,5}$ = 6.3 Hz, CH_3-5). ^{1}H NMR spectrum is in good agreement with literature [21].

Diphenyl (2,3,4-tri-*O*-acetyl-α-L-rhamnopyranosyl) phosphate (5α) and Diphenyl (2,3,4-tri-*O*-acetyl-β-L-rhamnopyranosyl) phosphate (5β)

2,3,4-Tri-*O*-acetyl-L-rhamnopyranose (**4**) (1 g, 3.45 mmol) was converted into an anomeric mixture of the corresponding diphenyl phosphates following a described procedure [14]. The mixture was separated using silica gel column chromatography (gradient of AcOEt with Et_3N (1%) against hexane with Et_3N (1%)) to give pure **5α** (102.7 mg, 6%) and **5β** (889.3 mg, 49%). Data for **5α**: $[\alpha]_D$ -41.8 (*c* 1.00, $CHCl_3$); Lit.[14]: $[\alpha]_D$ -41.3 (*c* 0.97, $CHCl_3$); $[\alpha]_D$ -45.6 (*c* 1.00, toluene). R_f = 0.65 (AcOEt-hexane 1:1 with Et_3N 1% v/v). ^{1}H NMR (toluene-d8, 400 MHz): δ_H 7.34-6.82 (10H, m, arom), 6.04 (1H, dd, $^3J_{1,P}$ = 6.7 Hz, $^3J_{1,2}$ = 1.3 Hz, H1), 5.56-5.52 (2H, m, H2, H3), 5.36 (1H, dd, $^3J_{3,4}$ = $^3J_{4,5}$ = 9.8 Hz, H4), 4.01 (1H, dq, $^3J_{CH3,5}$ = 6.1 Hz, H5), 1.70 (3H, s, OAc), 1.65 (3H, s, OAc), 1.55 (3H, s, OAc), 1.05 (3H, d, CH_3-5). ^{13}C NMR (toluene-d8, 100 MHz): δ_C 170.1, 169.8 (s, 3C, 3 x CH_3(*C*O)O), 151.8 (ds, 1C, $^2J_{Carom,P}$ = 6.49 Hz, C_{arom}), 151.5 (ds, 1C, $^2J_{Carom,P}$ = 7.05 Hz, C_{arom}), 130.9-121.2 (d, 10C, CH_{arom} overlapping with solvent signals), 97.6 (dd, 1C, $^2J_{C1,P}$ = 5.39 Hz, C1), 71.0 (d, 1C, C4), 70.1 (dd, 1C, $^3J_{C2,P}$ = 11.5 Hz, C2), 70.0 (d, 1C, C5), 69.6 (d, 1C, C3), 20.8, 20.6 (q, 3C, 3 x *C*H_3(CO)O), 18.0 (q, 1C, CH_3-

5). Proton-coupled ^{13}C NMR (toluene-d8, 100 MHz): $^{1}J_{C1,H1}$ = 179.4 Hz. ^{31}P NMR (toluene-d8, 162 MHz): δ_P -13.4. Data for **5β**: $[\alpha]_D$ +11.5 (*c* 1.00, $CHCl_3$); Lit.[14]: $[\alpha]_D$ +10.9 (*c* 0.99, $CHCl_3$); $[\alpha]_D$ -5.8 (*c* 1.00, toluene). R_f = 0.58 (AcOEt-hexane 1:1 with Et_3N 1% v/v). ^{1}H NMR (toluene-d8, 400 MHz): δ_H 7.34-6.83 (10H, m, arom), 5.60 (1H, bd, $^{3}J_{2,3}$ = 3.3 Hz, H2), 5.46 (1H, dd, $^{3}J_{1,P}$ = 6.9 Hz, $^{3}J_{1,2}$ = 1.2 Hz, H1), 5.22 (1H, dd, $^{3}J_{3,4}$ = 10.0 Hz, $^{3}J_{4,5}$ = 9.7 Hz, H4), 5.02 (1H, dd, H3), 3.13 (1H, dq, $^{3}J_{CH3,5}$ = 6.2 Hz, H5), 1.70 (3H, s, OAc), 1.68 (3H, s, OAc), 1.64 (3H, s, OAc), 1.03 (3H, d, CH_3-5). ^{13}C NMR (toluene-d8, 100 MHz): δ_C 170.3, 169.9 (s, 3C, 3 x $CH_3(CO)O$), 152.0 (ds, 1C, $^{2}J_{Carom,P}$ = 7.31 Hz, C_{arom}), 151.5 (ds, 1C, $^{2}J_{Carom,P}$ = 7.18 Hz, C_{arom}), 130.7-121.6 (d, 10C, CH_{arom} overlapping with solvent signals), 96.0 (dd, 1C, $^{2}J_{C1,P}$ = 4.3 Hz, C1), 71.9 (d, 1C, C5), 71.5 (dd, 1C, $^{4}J_{C3,P}$ = 1.1 Hz, C3), 71.2 (d, 1C, C4), 69.9 (dd, 1C, $^{3}J_{C2,P}$ = 9.4 Hz, C2), 20.8, 20.7 (q, 3C, 3 x $CH_3(CO)O$), 17.9 (q, 1C, CH_3-5). Proton-coupled ^{13}C NMR (toluene-d8, 100 MHz): $^{1}J_{C1,H1}$ = 165.7 Hz. ^{31}P NMR (toluene-d8, 162 MHz): δ_P -13.2.

β-L-Rhamnopyranosyl phosphate bistriethylammonium salt (6)

Diphenyl (2,3,4-tri-*O*-acetyl-β-L-rhamnopyranosyl) phosphate (**5β**) (712 mg, 1.36 mmol) was converted into **6** (556.1 mg, 91%) as described by Sabesan and Neira [14]. ^{1}H NMR (D_2O, 400 MHz, internal acetone at δ_H 2.22 ppm): δ_H 5.12 (1H, dd, $^{3}J_{1,P}$ = 8.6 Hz, $^{3}J_{1,2}$ = 1.0 Hz, H1), 4.01 (1H, bd, $^{3}J_{2,3}$ = 3.3 Hz, H2), 3.64 (1H, dd, $^{3}J_{3,4}$ = 9.4 Hz, H3), 3.43 (1H, dq, $^{3}J_{CH3\text{-}5,5}$ = 5.9 Hz, H5), 3.37 (1H, dd, $^{3}J_{4,5}$ = 9.4 Hz, H4), 3.20 (12H, q, $^{3}J_{CH3,CH2}$ = 7.3 Hz, 2 x $(CH_3CH_2)_3N$), 1.31 (3H, d, CH_3-5), 1.27 (18H, t, 2 x $(CH_3CH_2)_3N$). ^{13}C NMR (D_2O, 100 MHz, internal acetone): δ_C 95.8 (dd, 1C, $^{2}J_{C1,P}$ = 3.9 Hz, C1), 73.3 (d, 1C, C5), 72.9 (d, 1C, C3), 72.3 (d, 1C, C4), 71.7 (dd, 1C, $^{3}J_{C2,P}$ = 6.1 Hz, C2), 47.3 (t, 6C, 2 x $(CH_3CH_2)_3N$), 17.4 (q, 1C, CH_3-5), 8.9 (q, 6C, 2 x $(CH_3CH_2)_3N$). ^{31}P NMR (D_2O, 162 MHz): δ_P -1.6. ^{1}H, ^{13}C and ^{31}P NMR spectra are in good agreement with literature [14].

An analytical sample (15 mg) of **6** was converted into β-L-rhamnopyranosyl phosphate dicyclohexylammonium salt as described in literature [6]. $[\alpha]_D$ +12.1 (*c* 1.00, H_2O); Lit [14]. $[\alpha]_D$ +13.8 (*c* 1.00, H_2O); Lit [6]. $[\alpha]_D$ +11.9 (*c* 1.00, H_2O). Lit [13]. $[\alpha]_D$ +11.4 (*c* 0.5, H_2O).

Thymidine 5'-diphospho-β-L-rhamnose bistriethylammonium salt (1)

In order to remove traces of water, β-L-Rhamnopyranosyl phosphate bistriethylammonium salt (**6**) (29.6 mg, 66.3 μmol) and TMP-morpholidate (45.4 mg, 66.3 μmol) were separately dissolved in absolute pyridine (2 x 5 ml) and the solutions were evaporated to dryness. The compounds were re-dissolved in absolute pyridine (5 ml each) and the combined solutions were stirred at 4 °C under N_2 atmosphere. The reaction progress was monitored by SAX HPLC on Poros HQ 50 (see General Method A, TDP-β-L-rhamnose elutes at R_f = 4.8 min). After 5

days the conversion reached about 55%. The solvent was evaporated *in vacuo*, the residue was dissolved in Milli-Q water (2 ml) and freeze-dried. The crude mixture was re-dissolved in MeOH (2 ml), filtered through 0.45 µm disc filter and purified first by gel filtration on Sephadex LH-20 (see General Method B) followed by C18-RP chromatography (see General Method C, TDP-β-L-rhamnose eluted at R_f = 20.0 min). The bistriethylammonium salt of the pure sugar nucleotide **1** was obtained after freeze-drying as a white solid (6.3 mg, 16%). ^{1}H NMR (D_2O, 400 MHz): δ_H 7.67 (1H, d, $^4J_{6,CH3-5}$ = 1.0 Hz, H6), 6.27 (1H, t, $^3J_{1',2'}$ = 7.0 Hz, H1'), 5.14 (1H, dd, $^3J_{1'',P\beta}$ = 8.7 Hz, $^3J_{1'',2''}$ = 0.5 Hz, H1''), 4.57-4.53 (1H, m, H3'), 4.11-4.08 (3H, m, H4', H5'a, H5'b), 4.01 (1H, bd, $^3J_{2'',3''}$ = 3.2 Hz, H2''), 3.56 (1H, dd, $^3J_{3'',4''}$ = 9.4 Hz, H3''), 3.41-3.33 (1H, m, H5''), 3.30 (1H, dd, $^3J_{4'',5''}$ = 9.5 Hz, H4''), 3.12 (12H, q, $^3J_{CH2,CH3}$ = 7.3 Hz, $(CH_3C\mathit{H_2})_3N$), 2.35-2.24 (2H, m, H2'a, H2'b), 1.85 (3H, d, CH_3-5), 1.23 (3H, d, $^3J_{CH3,5''}$ = 6.0 Hz, CH_3-5''), 1.20 (18H, t, $(C\mathit{H_3}CH_2)_3N$). ^{1}H NMR spectrum in good agreement with literature [1-3]. ^{31}P NMR (D_2O, 162 MHz): δ_P -11.6 (d, 1P, $J_{P\alpha,P\beta}$ = 20.8 Hz, P_β), -13.7 (d, 1P, $J_{P\alpha,P\beta}$ = 20.9 Hz, P_α). ESIMS: *m/z* Calcd $[C_{16}H_{26}N_2O_{15}P_2 - H]^-$: 547.0736. Found: 547.0739.

Uridine 5'-diphospho-β-L-rhamnose bistriethylammonium salt (2)

In order to remove traces of water, β-L-rhamnopyranosyl phosphate bistriethylammonium salt (**6**) (50 mg, 112 µmol) and UMP-morpholidate (77 mg, 112 µmol) were separately dissolved in absolute pyridine (2 x 5 ml) and the solutions were evaporated to dryness. The compounds were re-dissolved in absolute pyridine (5 ml each) and the combined solutions were stirred at 4 °C under N_2 atmosphere. The reaction progress was monitored by SAX HPLC on Poros HQ 50 (see General Method A, UDP-β-L-rhamnose elutes at R_f = 5.3 min). After 7 days the conversion reached about 70%. The solvent was evaporated *in vacuo*, the residue was dissolved in Milli-Q water (2 ml) and freeze-dried. The crude mixture was re-dissolved in MeOH (2 ml), filtered through 0.45 µm disc filter and purified first by gel filtration on Sephadex LH-20 (see General Method B) followed by C18-RP chromatography (see General Method C, UDP-β-L-rhamnose eluted at R_f = 8.0 min). The bistriethylammonium salt of the pure sugar nucleotide **2** was obtained after freeze-drying as a white solid (12.8 mg, 21%). ^{1}H NMR (D_2O, 400 MHz, internal acetone at δ_H 2.22 ppm): δ_H 7.95 (1H, d, $^3J_{5,6}$ = 8.1 Hz, H6), 5.99 (1H, d, $^3J_{1',2'}$ = 4.4 Hz, H1'), 5.97 (1H, d, H5), 5.22 (1H, bd, $^3J_{1'',P\beta}$ = 8.8 Hz, $^3J_{1'',2''}$ < 1 Hz, H1''), 4.38-4.37 (2H, m, H2', H3'), 4.30-4.26 (1H, m, H4'), 4.23-4.16 (2H, m, H5'a, H5'b), 4.09 (1H, bd, $^3J_{2'',3''}$ = 3.3 Hz, H2''), 3.64 (1H, dd, $^3J_{3'',4''}$ = 9.5 Hz, H3''), 3.48-3.36 (1H, m, H5''), 3.37 (1H, dd, $^3J_{4'',5''}$ = 9.8 Hz, H4''), 3.20 (12H, q, $^3J_{CH2,CH3}$ = 7.3 Hz, $(CH_3C\mathit{H_2})_3N$), 1.31 (3H, d, $^3J_{CH3,5''}$ = 6.0 Hz, CH_3-5''), 1.27 (18H, t, $(C\mathit{H_3}CH_2)_3N$). ^{1}H NMR spectrum in good agreement with literature [12,13]. ^{13}C NMR (D_2O, 100 MHz, internal acetone): δ_C 166.9 (s, 1C, C4), 152.5 (s, 1C, C2),

142.3 (d, 1C, C6), 103.4 (d, 1C, C5), 96.2 (dd, 1C, ${}^{2}J_{C1'',P\beta}$ = 3.68 Hz, C1''), 89.0 (d, 1C, C1'), 83.9 (dd, 1C, ${}^{3}J_{C4',P\alpha}$ = 9.07 Hz, C4'), 74.4 (d, 1C, C2'), 73.4 (d, 1C, C5''), 72.8 (d, 1C, C3''), 72.3 (d, 1C, C4''), 71.5 (dd, 1C, ${}^{3}J_{C2'',P\beta}$ = 5.80 Hz, C2''), 70.3 (d, 1C, C3'), 65.6 (dt, 1C, ${}^{2}J_{C5',P\alpha}$ = 5.15 Hz, C5'), 47.3 (t, 6C, 2 x $(CH_3\mathit{C}H_2)_3N$), 17.4 (q, 1C, CH_3-5''), 8.9 (q, 6C, 2 x $(\mathit{C}H_3CH_2)_3N$). ^{31}P NMR (D_2O, 162 MHz): δ_P -11.4 (d, 1P, $J_{P\alpha,P\beta}$ = 17.4 Hz, P_β), -13.6 (d, 1P, $J_{P\alpha,P\beta}$ = 17.4 Hz, P_α). ^{31}P NMR spectrum is in good agreement with literature [13].

Sugar nucleotide extraction and profiling

Pelleted cells of *E. gracilis* and *P. parvum* containing known amount of the appropriate internal standards were lysed with cold (-20 °C) 70% ethanol (20 ml) in an ice bath for 1 h with occasional shaking / vortexing. The cell debris was removed by centrifugation (28928 x *g*, 20 min, 4 °C) and the supernatant was transferred into a glass round-bottom flask (100 ml). Ethanol was evaporated at reduced pressure and ambient temperature and the aqueous residue was freeze dried. At this stage the sample can be stored at -80 °C for any length of time before the next step.

Lipophilic components were removed by partitioning the sample between water and butan-1-ol [22]. In brief, the sample was dissolved in 9% aqueous butan-1-ol (3 x 2 ml) and transferred into a glass vial (10 ml volume). The solution was extracted with 90% butan-1-ol (3 x 2 ml) to remove the top layer containing lipids (but also chlorophyll, and insoluble polysaccharides such as paramylon forming middle layer). The bottom layer was collected and extracted again. Centrifugation was used to speed up the separation of the layers (200 - 800 x *g*, 4 °C, 5 min). The clear aqueous layer was collected and freeze dried in a pear-shaped flask (foaming may appear under vacuum). Samples were stored at -80 °C before the next step.

Solid phase extraction (SPE) of sugar nucleotides was performed essentially as described by Rabina and co-workers [23]. In brief, a graphitised carbon column (EnviCarb, Supelco, 250 mg, 3 ml) was conditioned by washing with 80% aqueous acetonitrile containing 0.1 % trifluoroacetic acid (3 ml) followed by water (2 ml). The sample was dissolved in ammonium bicarbonate (5 mM, 500 µl) and applied on the SPE column. The column was washed with water (2 ml), followed by 25% aqueous acetonitrile (2 ml), and 50 mM triethylammonium acetate buffer (pH 7.0, 2 ml). Finally, the sugar nucleotides were eluted with 50 mM triethylammonium acetate buffer pH 7.0 containing 25% acetonitrile (1.5 ml). The sample was filtered using 0.45 µm disc filters (PTFE) and freeze dried. Samples were stored at -80 °C prior to LC-MS/MS analysis.

LC-MS/MS profiling of sugar nucleotides was performed on a Xevo TQ-S tandem quadrupole mass spectrometer (Waters) operated in multiple reaction monitoring (MRM) mode coupled to

an Acquity UPLC. ESI-MS/MS analysis was performed in negative ion mode using a source with a capillary voltage of 1.5 kV, 500 °C desolvation temperature, 1000 $l.hr^{-1}$ desolvation gas, 150 $l.hr^{-1}$ cone gas, and 7 bar nebulizer pressure. MRM transitions for sugar nucleotide standards in negative ESI mode were generated using IntelliStart software (Table S1). Samples (10 µM) were introduced at 10 µl/min combined with a flow from the HPLC pump typical of an LC run. Once LC retention times of standards have been established, the mass transitions were collected in time-windows centred on the relevant peaks, to avoid collecting excessive numbers of transitions simultaneously. MassLynx software (Waters) was used to collect, to analyse and to process data.

Liquid chromatography separation of sugar nucleotides was achieved on a surface-conditioned PGC column (Hypercarb, Thermo Scientific, dimensions 1 x 100 mm, particle size 5 µm) equipped with a column guard (Hypercarb, 5 µm, 1 x 10 mm). Sugar nucleotides were eluted using mobile phase A: formic acid 0.3% brought to pH 9.0 with ammonia and mobile phase B: acetonitrile using the following multistep gradient at a flow rate 80 µl/min: 0 min: 2% B; 20 min: 15% B; 26 min: 50% B; 27 min: 90% B; 30 min: 90% B; 31 min: 2% B; 50 min: 2% B. Available sugar nucleotide standards (10 µM) were injected (5 µl) to determine retention times (Table S1).

Limit of detection was determined to be 10 fmol on column using a serial dilution of UDP-α-D-Glc. Samples of extracted sugar nucleotides were reconstituted in buffer A (25 µl) and injected (5 µl, 20 % of total) using partial loop injection. Analysis of 3 biological replicates was performed. Where in doubt, co-injection of sample with appropriate standard sugar nucleotide was used for positive identification. Data processing was performed using MassLynx (Waters) software. Although between runs there were significant differences in absolute retention times of standards, relative retentions were reasonably reproducible (Table S1). To ensure maximum retention time (R_f) stability, after a batch of samples, the PGC column had to be regenerated and its performance was tested using UDP-α-D-Glc as a standard. The regeneration and column performance steps were performed using a standard HPLC system (Ultimate 3000, Dionex) with UV detection at 265 nm. Column performance was tested first before regeneration steps at flow rate 80 µl/min by injecting UDP-α-D-Glc (5 µl, 10 µM) standard and elution using mobile phases A and B as mentioned earlier. The column was then washed for 3 hrs with mobile phase C (acetonitrile 80%, water 20 %, TFA 0.1%) [24] followed by water (5 column volumes).

Next, the PGC column was reduced at flow rate 80 µl/min with freshly prepared sodium sulphite (100 mM) for 24 h [25] followed by MQ water (5 column volumes). The column was then washed at 80 µl/min with high acetonitrile (90% B, 10% A) for 30 min and equilibrated

with 2% B, 98 % A for 10 min. The column performance was tested again by injecting UDP-α-D-Glc (5 µl, 10 µM) standard. The column was stored in 2% B, 98 % A

**Sugar Nucleotide**	**Relative Retention time**	**MRM transitions**	**Fragment**
UDP-α-D-Glc	1.00	565 → 323<br>565 → 79	$[NMP-H]^-$<br>$[H_3PO_4-H_3O]^-$
UDP-α-D-GlcNAcA	0.89	620 → 403<br>620 → 159	$[NDP-H]^-$<br>$[H_4P_2O_7-H_3O]^-$
UDP-β-L-Rha	0.84	549 → 323<br>549 → 159	$[NMP-H]^-$<br>$[H_4P_2O_7-H_3O]^-$
TDP-α-D-Glc	1.39	563 → 321<br>563 → 241	$[NMP-H]^-$<br>$[Glc-1-P-H-H_2O]^-$
TDP-β-L-Rha	1.35	547 → 321<br>547 → 225	$[NMP-H]^-$<br>$c[Rha-1-P-H-H_2O]^-$
GDP-α-D-Glc	1.56	604 → 362<br>604 → 241	$[NMP-H]^-$<br>$c[Glc-1-P-H-H_2O]^-$

Table S1– Relative retention times and MRM transitions of sugar nucleotides examined in this study.

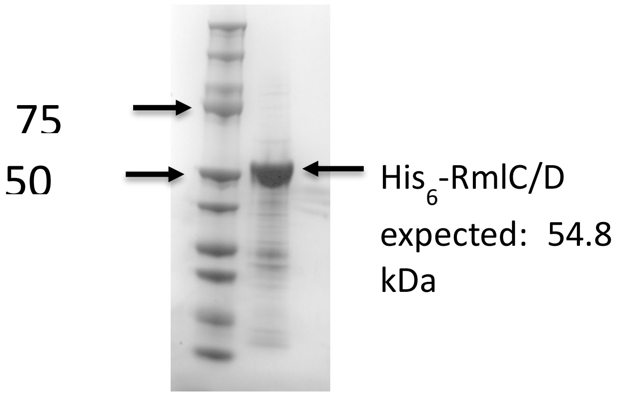

Figure S2 - SDS-PAGE analysis of Nickel column purified *P. parvum* RmlCD produced in *E. coli* in this study.

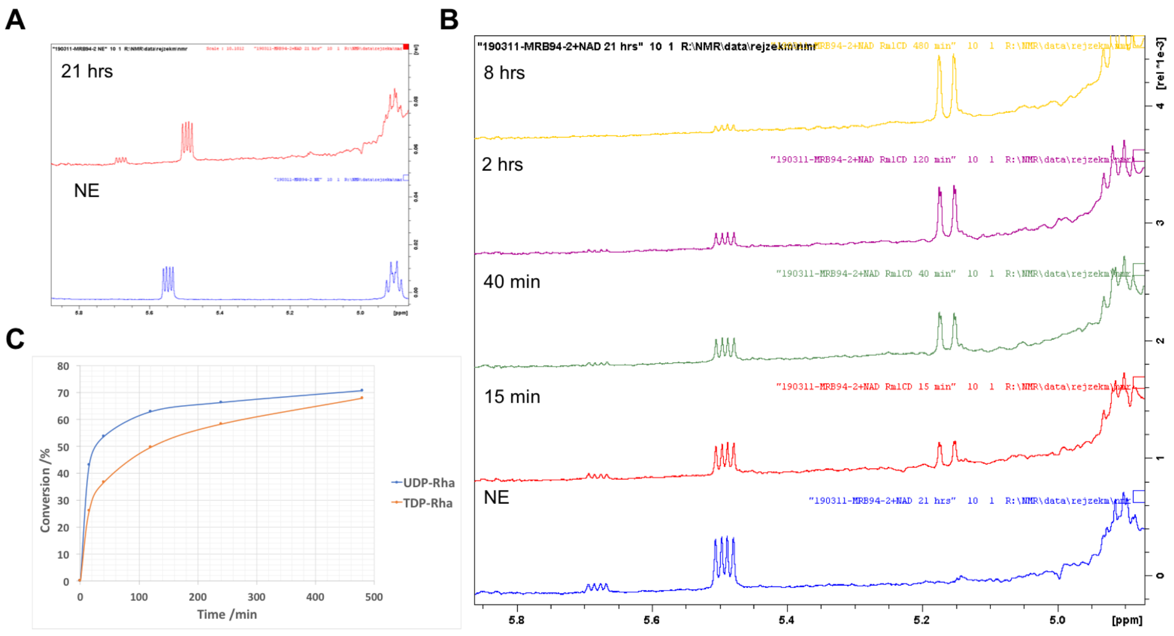

Figure S3 - Production of UDP-β-L-Rha using ATCV-1 UDP-glucose 4,6-dehydratase and *P. parvum* RmlCD. (A) ^{1}H NMR showing complete conversion of UDP-α-D-Glc to UDP-6-deoxy-α-D-*xylo*-hexos-4-ulose after 21 hours of incubation with ATCV-1 UGD. Loss of anomeric proton signal of UDP-α-D-Glc (~5.55 ppm) and formation of 2 new signals representing the keto (~5.68 ppm) and hydrated form (~5.48 ppm) of UDP-6-deoxy-α-D-*xylo*-hexos-4-ulose is monitored. (B) ^{1}H NMR time course showing formation of UDP-β-L-Rha over an 8-hour time course mediated by *P. parvum* RmlCD. Loss of anomeric signals representing UDP-6-deoxy-α-D-*xylo*-hexos-4-ulose and formation of a new signal corresponding to the anomeric proton of UDP-β-L-Rha (~5.16 ppm) can be observed. (C) Relative rates of conversion at various timepoints observed for RmlCD when producing UDP-β-L-Rha and TDP-β-L-Rha.

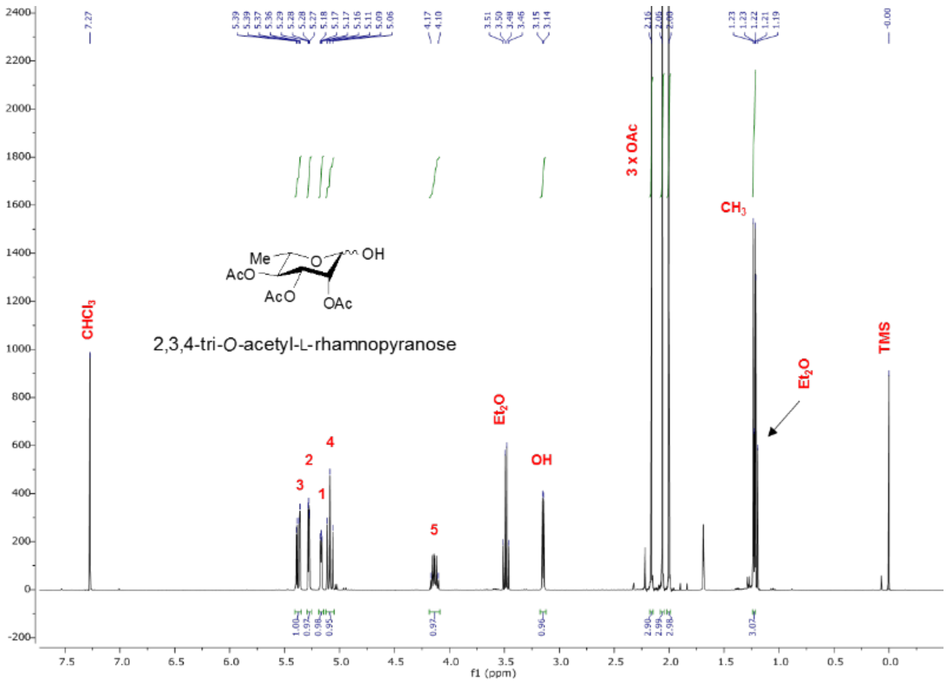

Figure S4 - ^{1}H NMR spectrum ($CDCl_3$, 400 MHz) of 2,3,4-tri-*O*-acetyl-L-rhamnopyranose (4).

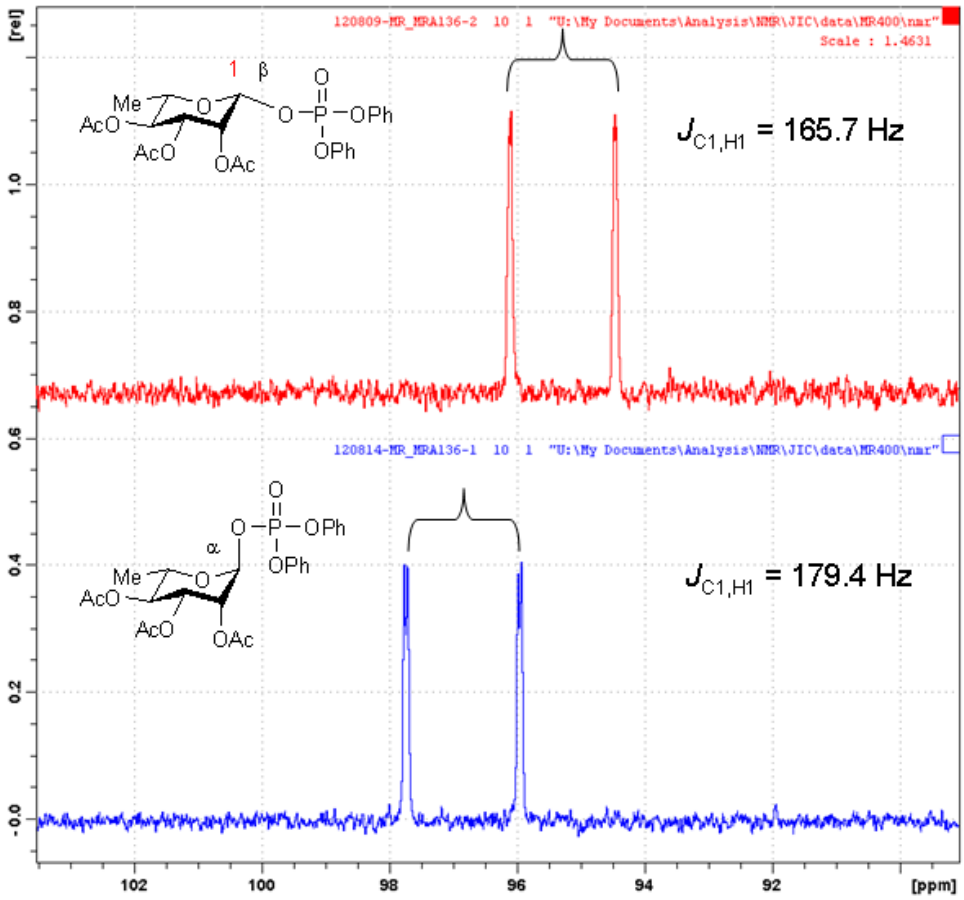

Figure S5 – ^{1}H coupled ^{13}C NMR of 5β and 5α in toluene-d8.

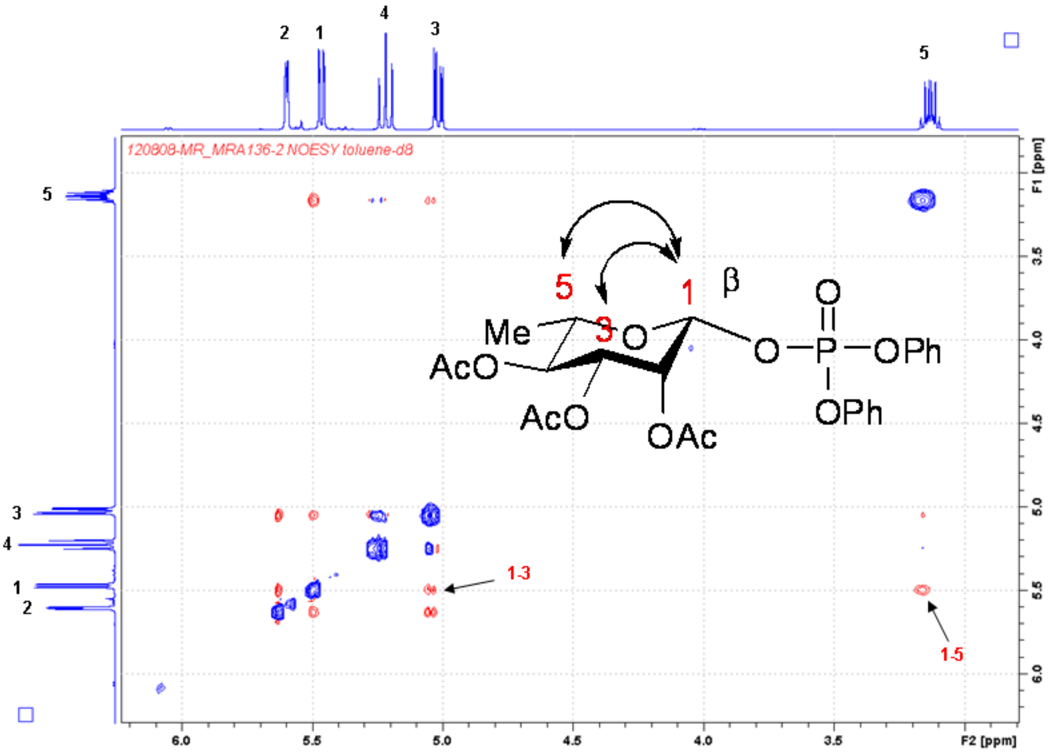

Figure S6 – NOESY spectrum of 5β in toluene-d8.

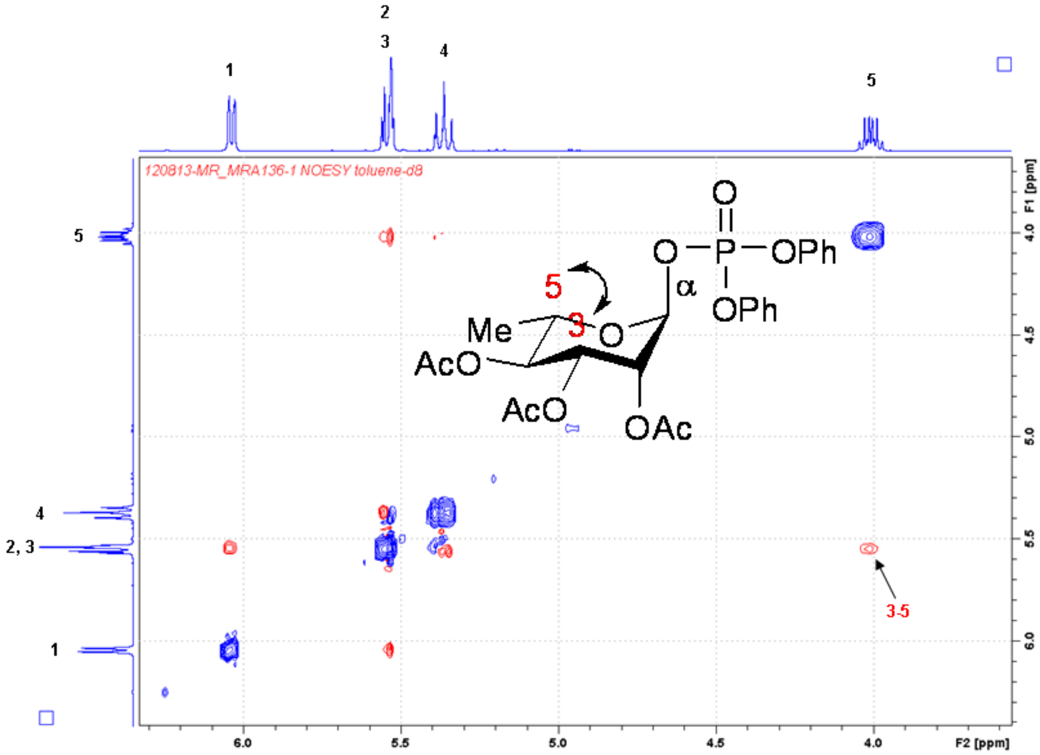

Figure S7 – NOESY spectrum of 5α in toluene-d8.

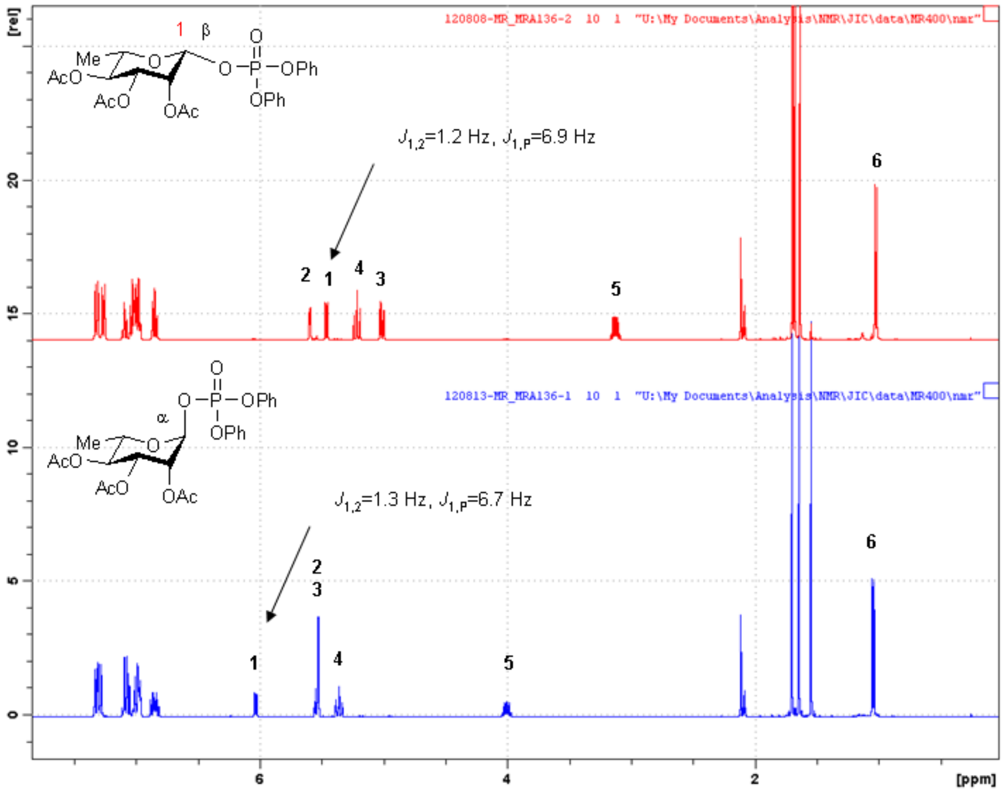

Figure S8 – Stacked ^{1}H NMR spectra of 5α and 5β in toluene-d8.

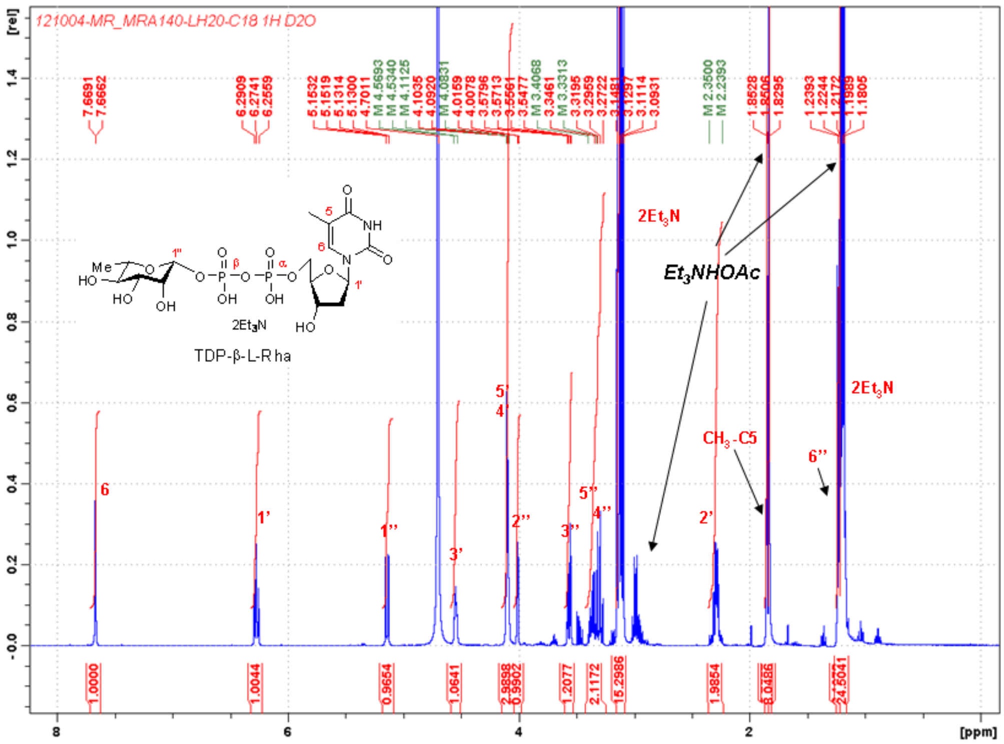

Figure S9 – ^{1}H NMR spectrum (D_2O, 400 MHz) of TDP-β-L-Rhamnose bistriethylammonium salt (1).

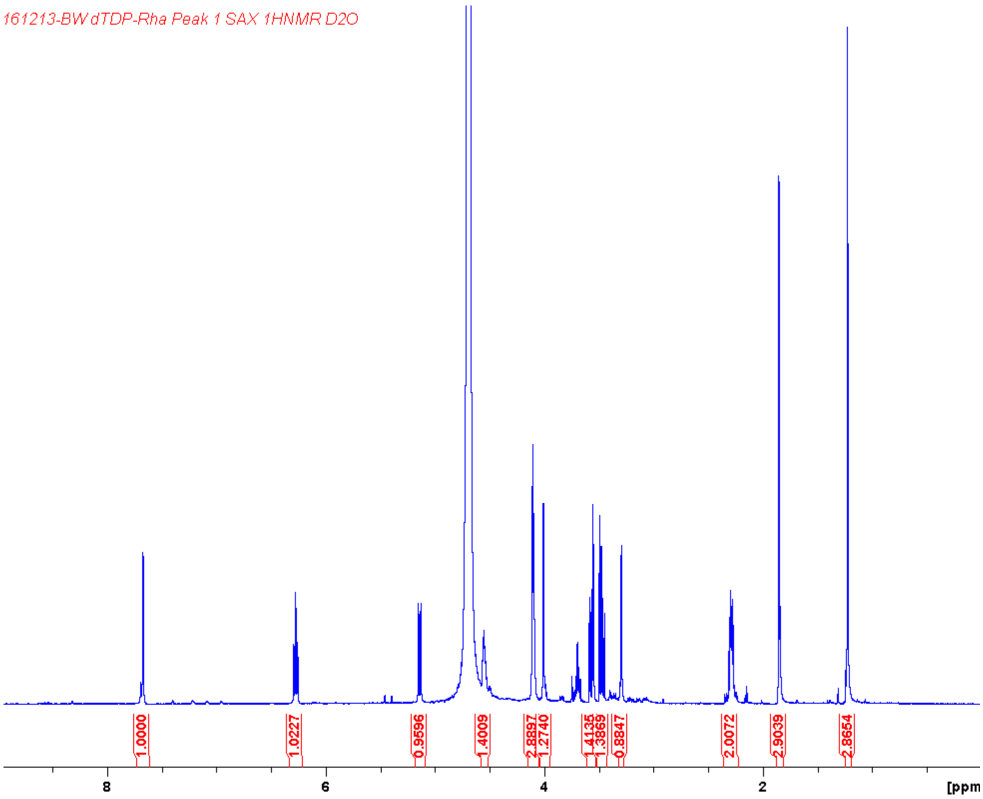

Figure S10 - ^{1}H NMR spectrum (D_2O, 400 MHz) of TDP-β-L-Rhamnose produced enzymatically in this study.

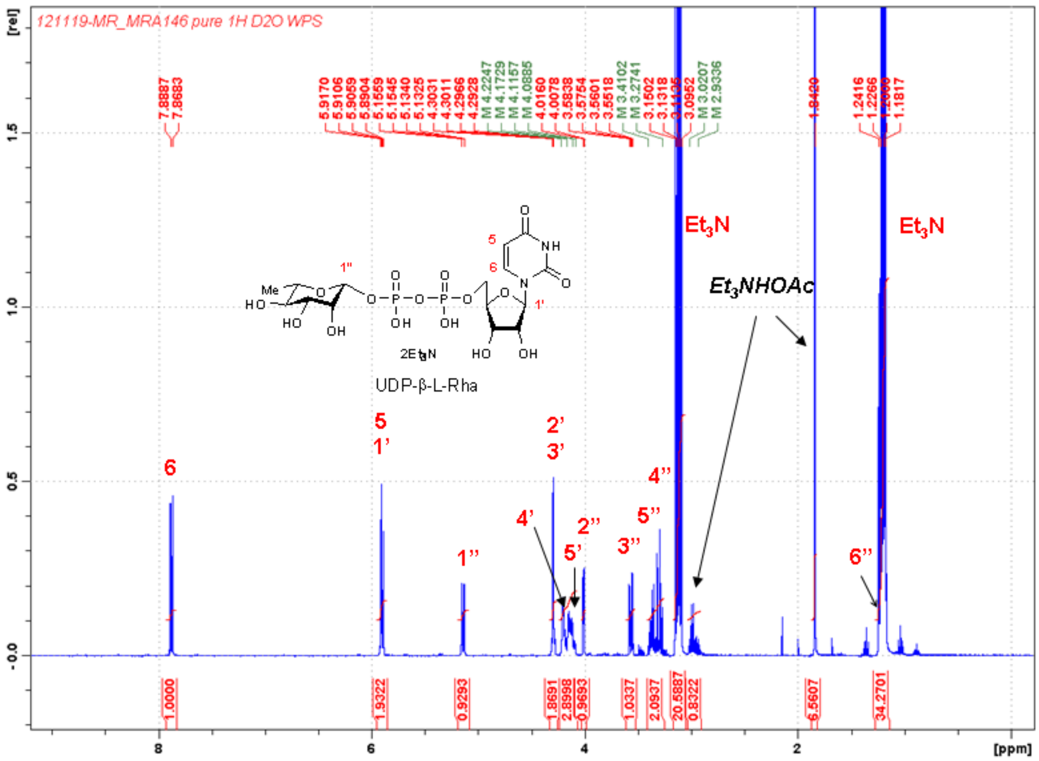

Figure S2 – ^{1}H NMR spectrum (D_2O, 400 MHz, internal acetone at δ_H 2.22 ppm) of UDP-β-L-Rhamnose bistriethylammonium salt (2).

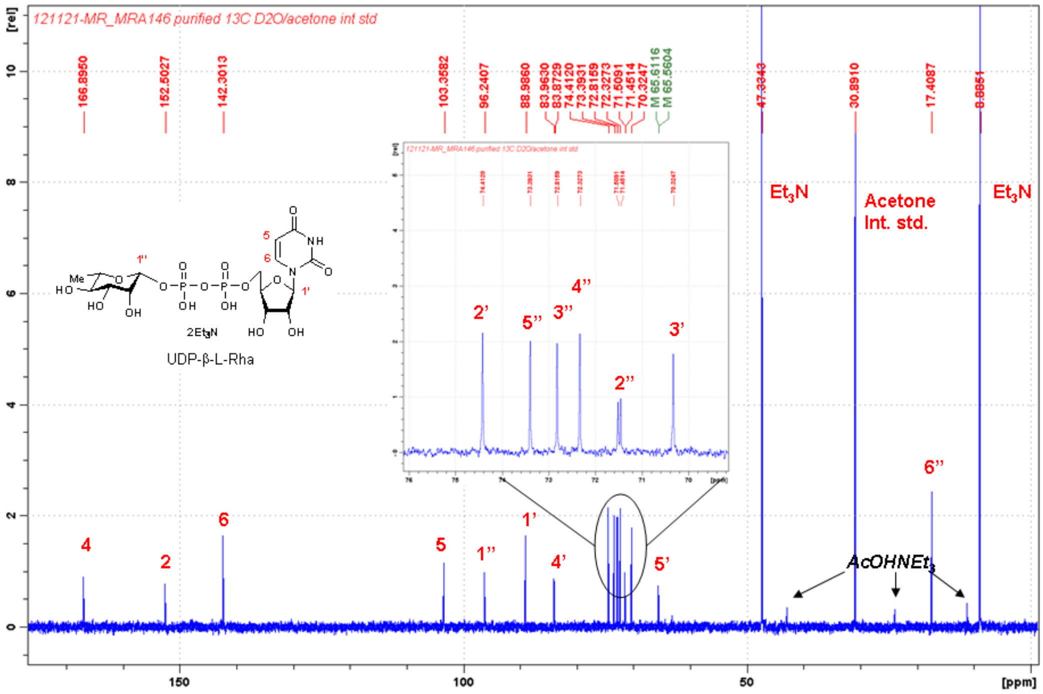

Figure S3 - ^{13}C NMR spectrum (D_2O, 100 MHz, internal acetone at δ_C 30.89 ppm) of UDP-β-L-Rhamnose bistriethylammonium salt (2).

Kingdom	Phylum	Class	Family	Genus	Species	Strain	Database and identifier	Sequence
**GLAUCOPHYTES**	Glaucophyta	Glaucocystophyceae	Glaucosphaeraceae	*Cyanoptyche*	*gloeocystis*	SAG4.97	MMETSP1086	CAMPEP_0196658872
	Glaucophyta	Glaucophyceae	Gloeochaetaceae	*Gloeochaete*	*wittrockiana*	SAG46.84	MMETSP1089	CAMPEP_0184350920, CAMPEP_0184349520
**GREEN ALGAE**	Chlorophyta	Chlorodendrophyceae	Chlorodendraceae	*Tetraselmis*	*striata*	LANL1001	MMETSP0817, MMETSP0818, MMETSP0819, MMETSP0820	CAMPEP_0200914898
	Chlorophyta	Chlorophyceae	Dunaliellaceae	*Dunaliella*	*tertiolecta*	CCMP1320	MMETSP1126, MMETSP1127, MMETSP1128	CAMPEP_0187373064
	Chlorophyta	Chlorophyceae	Chlamydomonadaceae	*Chlamydomonas*	*reinhardtii*	CC-503 cw92 mt+	https://www.ncbi.nlm.nih.gov/nuccore/158276217	XP_001695032.1
	Chlorophyta	Mamiellophyceae	Mamiellaceae	*Micromonas*	sp.	RCC472	MMETSP1084, MMETSP1387	
	Chlorophyta	Mamiellophyceae	Mamiellaceae	*Micromonas*	sp.	NEPCC29	MMETSP1386, MMETSP1082	
	Chlorophyta	Mamiellophyceae	Mamiellaceae	*Micromonas*	sp.	CCMP2099	MMETSP1390, MMETSP0802	CAMPEP_0190189836
	Chlorophyta	Mamiellophyceae	Mamiellaceae	*Micromonas*	*pusilla*	CCMP1545	https://www.ncbi.nlm.nih.gov/genome/?term=txid564608[Organism:noexp]	XP_003057320.1
	Chlorophyta	Mamiellophyceae	Mamiellaceae	*Bathycoccus*	*prasinos*	RC1105	https://www.ncbi.nlm.nih.gov/genome/12309	XP_007514013.1, XP_007508076.1

	Chlorophyta	Mamiellophyceae	Mamiellaceae	*Ostreococcus*	*tauri*		https://www.ncbi.nlm.nih.gov/genome/373	CEG01892.1
	Chlorophyta	Pyramimonadophyceae	Halosphaeraceae	*Pyramimonas*	*parkeae*	CCMP726	MMETSP0058, MMETSP0059	CAMPEP_0191497618, CAMPEP_0191502286
	Chlorophyta	Trebouxiophyceae	Chlorellaceae	*Auxenochlorella*	*protothecoides*	sp 0710	http://www.ncbi.nlm.nih.gov/genome?LinkName=nuccore_genome&from_uid=667612142	XP_011395817.1
	Chlorophyta	Trebouxiophyceae	Chlorellaceae	*Chlorella*	*variabilis*	NC64A	https://www.ncbi.nlm.nih.gov/genome/694	XP_005851985.1
	Chlorophyta	Unknown	Unknown	*Picocystis*	*salinarum*	CCMP1897	MMETSP1159, MMETSP0807	CAMPEP_0190751704
**RED ALGAE**	Rhodophyta	Compsopogonophyceae	Compsopogonaceae	*Compsopogon*	*coeruleus*	SAG 36.94	MMETSP0312	
	Rhodophyta	Compsopogonophyceae	Erythrotrichiaceae	*Madagascaria*	*erythrocladiodes*	CCMP3234	MMETSP1450	CAMPEP_0198320684
	Rhodophyta	Cyanidiophyceae	Galdieriaceae	*Galdieria*	*sulphuraria*	074W	http://www.ncbi.nlm.nih.gov/genome/405	XP_005707655.1
	Rhodophyta	Florideophyceae	Gigartinaceae	*Chondrus*	*crispus*	Stackhouse	http://www.ncbi.nlm.nih.gov/genome/12106	
	Rhodophyta	Porphyridiophyceae	Porphyridiaceae	*Erythrolobus*	*australicus*	CCMP3124	MMETSP1353	
	Rhodophyta	Porphyridiophyceae	Porphyridiaceae	*Erythrolobus*	*madagascarensis*	CCMP3276	MMETSP1354	

	Rhodophyta	Porphyridiophyceae	Porphyridiaceae	*Timspurckia*	*oligopyrenoides*	CCMP3278	MMETSP1172	
	Rhodophyta	Rhodellophyceae	Porphyridiaceae	*Porphyridium*	*aerugineum*	SAG 1380-2	MMETSP0313	
	Rhodophyta	Rhodellophyceae	Rhodellaceae	*Rhodella*	*maculata*	CCMP736	MMETSP0167, MMETSP0314	CAMPEP_0191515942, CAMPEP_0191515916
	Rhodophyta	Rhodellophyceae	Stylonemataceae	*Rhodosorus*	*marinus*	CCMP 769	MMETSP0011	
	Rhodophyta	Rhodellophyceae	Stylonemataceae	*Rhodosorus*	*marinus*	UTEX LB 2760	MMETSP0315	
**EXCAVATES**	Euglenozoa	Euglenophyceae	Eutreptiaceae	*Eutreptiella*	*gymnastica*	NIES-381	MMETSP0039	
	Euglenozoa	Euglenophyceae	Eutreptiaceae	*Eutreptiella*	*gymnastica-like*	CCMP1594	MMETSP0809, MMETSP0810, MMETSP0811	CAMPEP_0200405894
	Euglenozoa	Euglenophyceae	Euglenaceae	*Euglena*	*gracilis*	CCAP 1124/7a		light_m.86199
**RHIZARIA**	Cercozoa	Chlorarachniophyceae	Chlorarachniaceae	*Chlorarachnion*	*reptans*	CCCM449	MMETSP0109	CAMPEP_0114518010
	Cercozoa	Chlorarachniophyceae	Chlorarachniaceae	*Gymnochlora*	sp.	CCMP2014	MMETSP0110	CAMPEP_0167746174, CAMPEP_0167745746
	Cercozoa	Chlorarachniophyceae	Chlorarachniaceae	*Lotharella*	*oceanica*	CCMP622	MMETSP0040	CAMPEP_0170191836

	Cercozoa	Chlorarachniophyceae	Chlorarachniaceae	*Lotharella*	*globosa*	CCCM811	MMETSP0111, MMETSP0112	CAMPEP_0190181758
	Cercozoa	Chlorarachniophyceae	Chlorarachniaceae	*Bigelowiella*	*natans*	CCMP623	MMETSP1052	CAMPEP_0169532188, CAMPEP_0169546672,
	Cercozoa	Chlorarachniophyceae	Chlorarachniaceae	*Bigelowiella*	*natans*	CCMP 2755	MMETSP0045	CAMPEP_0114200706
	Cercozoa	Chlorarachniophyceae	Chlorarachniaceae	*Bigelowiella*	*natans*	CCMP1259	MMETSP1054	CAMPEP_0169599456
**CRYPTOPHYTES**	Cryptophyta	Cryptophyceae	Cryptomonadaceae	*Cryptomonas*	*paramecium*	CCAP977/2a	MMETSP0038	CAMPEP_0113696002
	Cryptophyta	Cryptophyceae	Cryptomonadaceae	*Cryptomonas*	*curvata*	CCAP979/52	MMETSP1050	CAMPEP_0172168610, CAMPEP_0172173114
	Cryptophyta	Cryptophyceae	Geminigeraceae	*Geminigera*	*cryophila*	CCMP2564	MMETSP0799	CAMPEP_0179481402, CAMPEP_0179458582
	Cryptophyta	Cryptophyceae	Geminigeraceae	*Geminigera*	sp.	Caron Lab Isolate	MMETSP1102	CAMPEP_0173087898, CAMPEP_0173097438
	Cryptophyta	Cryptophyceae	Geminigeraceae	*Guillardia*	*theta*	CCMP 2712	MMETSP0046	CAMPEP_0113812340
	Cryptophyta	Cryptophyceae	Hemiselmidaceae	*Hemiselmis*	*andersenii*	CCMP439	MMETSP1041	CAMPEP_0172041234
	Cryptophyta	Cryptophyceae	Hemiselmidaceae	*Hemiselmis*	*andersenii*	CCMP1180	MMETSP1042	CAMPEP_0169429296

	Cryptophyta	Cryptophyceae	Hemiselmidaceae	*Hemiselmis*	*andersenii*	CCMP441	MMETSP1043	CAMPEP_0172046830
	Cryptophyta	Cryptophyceae	Hemiselmidaceae	*Hemiselmis*	*tepida*	CCMP443	MMETSP1355	CAMPEP_0174941776
	Cryptophyta	Cryptophyceae	Hemiselmidaceae	*Hemiselmis*	*rufescens*	PCC563	MMETSP1357	CAMPEP_0173449620, CAMPEP_0173437524
	Cryptophyta	Cryptophyceae	Hemiselmidaceae	*Hemiselmis*	*viresens*	PCC157	MMETSP1356	CAMPEP_0173408906, CAMPEP_0173411170
	Cryptophyta	Cryptophyceae	Goniomonodaceae	*Goniomonas*	*Pacifica*	CCMP1869	MMETSP0107, MMETSP0108	CAMPEP_0188509280
	Cryptophyta	Cryptophyceae	Pyrenomonadaceae	*Rhodomonas*	*salina*	CCMP1319	MMETSP1047	CAMPEP_0172097468, CAMPEP_0172099434
	Cryptophyta	Cryptophyceae	Pyrenomonadaceae	*Rhodomonas*	sp.	CCMP768	MMETSP1091, MMETSP1389	CAMPEP_0191543004
	Cryptophyta	Cryptophyceae	Pyrenomonadaceae	*Rhodomonas*	*abbreviata*	Caron Lab Isolate	MMETSP1101	CAMPEP_0181338540, CAMPEP_0181298928, CAMPEP_0181313830, CAMPEP_0181297718
**HAPTOPHYTES**	Haptophyta	Pavlovophyceae	Pavlovaceae	*Pavlova*	*gyrans*	CCMP608	MMETSP1466	CAMPEP_0206039264, CAMPEP_0206053400
	Haptophyta	Pavlovophyceae	Pavlovaceae	*Pavlova*	*lutheri*	RCC1537	MMETSP1463	

	Haptophyta	Pavlovophyceae	Pavlovaceae	*Pavlova*	sp.	CCMP459	MMETSP1139, MMETSP1140, MMETSP1381	CAMPEP_0190524078,
	Haptophyta	Prymnesiophyceae	Prymnesiaceae	*Chrysochromulina*	*polylepis*	UIO037	MMETSP0286	
	Haptophyta	Prymnesiophyceae	Prymnesiaceae	*Chrysochromulina*	*polylepis*	CCMP1757	MMETSP0143, MMETSP0145, MMETSP0146, MMETSP0147	CAMPEP_0193719730, CAMPEP_0193726698
	Haptophyta	Prymnesiophyceae	Prymnesiaceae	*Chrysochromulina*	*rotalis*	UIO044	MMETSP0287	CAMPEP_0115854040
	Haptophyta	Prymnesiophyceae	Prymnesiaceae	*Chrysochromulina*	*ericina*	CCMP281	MMETSP1096	CAMPEP_0181246850, CAMPEP_0181201634,
	Haptophyta	Prymnesiophyceae	Prymnesiaceae	*Chrysochromulina*	*brevifilum*	UTEX LB 985	MMETSP1094	CAMPEP_0174727498
	Haptophyta	Prymnesiophyceae	Prymnesiaceae	*Prymnesium*	*parvum*	Texoma1	MMETSP0006, MMETSP0007, MMETSP0008, MMETSP0815, MMETSP0814	CAMPEP_0191228776,
	Haptophyta	Prymnesiophyceae	Noelaerhabdaceae	*Emiliania*	*huxleyi*	374	MMETSP1006, MMETSP1007, MMETSP1008, MMETSP1009	CAMPEP_0187581196
	Haptophyta	Prymnesiophyceae	Noelaerhabdaceae	*Emiliania*	*huxleyi*	379	MMETSP0994, MMETSP0995, MMETSP0996, MMETSP0997	CAMPEP_0187642360
	Haptophyta	Prymnesiophyceae	Noelaerhabdaceae	*Emiliania*	*huxleyi*	PLY M219	MMETSP1150, MMETSP1151, MMETSP1152, MMETSP1153	CAMPEP_0187777132
	Haptophyta	Prymnesiophyceae	Noelaerhabdaceae	*Emiliania*	*huxleyi*	CCMP370	MMETSP1154, MMETSP1155, MMETSP1156, MMETSP1157	CAMPEP_0187665496

	Haptophyta	Prymnesiophyceae	Noelaerhabdaceae	*Emiliania*	*huxleyi*	CCMP1516	http://www.ncbi.nlm.nih.gov/genome/2	XP_005785625.1
	Haptophyta	Prymnesiophyceae	Noelaerhabdaceae	*Gephyrocapsa*	*oceanica*	RCC1303	MMETSP1363, MMETSP1364, MMETSP1365, MMETSP1366	CAMPEP_0188174236
	Haptophyta	Prymnesiophyceae	Isochrysidaceae	*Isochrysis*	sp.	CCMP1324	MMETSP1129, MMETSP1130, MMETSP1131, MMETSP1132	CAMPEP_0188830390
	Haptophyta	Prymnesiophyceae	Isochrysidaceae	*Isochrysis*	sp.	CCMP1244	MMETSP1090, MMETSP1388	CAMPEP_0188768992
	Haptophyta	Prymnesiophyceae	Isochrysidaceae	*Isochrysis*	*galbana*	CCMP1323	MMETSP0944, MMETSP0943, MMETSP0595	CAMPEP_0193694840, CAMPEP_0193669402
	Haptophyta	Prymnesiophyceae	Phaeocystaceae	*Phaeocystis*	sp.	CCMP2710	MMETSP1162	
	Haptophyta	Prymnesiophyceae	Phaeocystaceae	*Phaeocystis*	*antarctica*	Caron Lab Isolate	MMETSP1100	CAMPEP_0172959570
	Haptophyta	Prymnesiophyceae	Phaeocystaceae	*Phaeocystis*	*antarctica*	CCMP1374	MMETSP1444	CAMPEP_0198174474
	Haptophyta	Prymnesiophyceae	Pleurochrysidaceae	*Pleurochrysis*	*carterae*	CCMP645	MMETSP1136, MMETSP1137, MMETSP1138	CAMPEP_0190806002
**STRAMENOPILES**	Ochrophyta	Bacillariophyceae	Amphipleuraceae	*Amphiprora*	sp.	CCMP467	MMETSP0725, MMETSP0726, MMETSP0727, MMETSP0724	CAMPEP_0186506366, CAMPEP_0186488760
	Ochrophyta	Bacillariophyceae	Catenulaceae	*Amphora*	*coffeaeformis*	CCMP127	MMETSP0316, MMETSP0317, MMETSP0318	CAMPEP_0186538782

	Ochrophyta	Bacillariophyceae	Bacillariaceae	*Fragilariopsis*	*kerguelen sis*	L26-C5	MMETSP0733, MMETSP0734, MMETSP0735, MMETSP0736	CAMPEP_0188139216
	Ochrophyta	Bacillariophyceae	Bacillariaceae	*Fragilariopsis*	*kerguelen sis*	L2-C3	MMETSP0906, MMETSP0907, MMETSP0908, MMETSP0909	
	Ochrophyta	Bacillariophyceae	Bacillariaceae	*Nitzschia*	*punctata*	CCMP561	MMETSP0744, MMETSP0745, MMETSP0746, MMETSP0747	CAMPEP_0199327438
	Ochrophyta	Bacillariophyceae	Bacillariaceae	*Pseudo-nitzschia*	*australis*	10249 10 AB	MMETSP0139, MMETSP0142, MMETSP0140, MMETSP0141	CAMPEP_0199647432
	Ochrophyta	Bacillariophyceae	Bacillariaceae	*Pseudo-nitzschia*	*fraudulent a*	WWA7	MMETSP0850, MMETSP0851, MMETSP0852, MMETSP0853	CAMPEP_0199791592
	Ochrophyta	Bacillariophyceae	Chaetocerotace ae	*Chaetoceros*	*debilis*	MM31A-1	MMETSP0149, MMETSP0150	CAMPEP_0200879214
	Ochrophyta	Bacillariophyceae	Chaetocerotace ae	*Chaetoceros*	*neogracile*	CCMP1317	MMETSP0751, MMETSP0752, MMETSP0753, MMETSP0754	CAMPEP_0201017622, CAMPEP_0201002392, CAMPEP_0200999620
	Ochrophyta	Bacillariophyceae	Chaetocerotace ae	*Chaetoceros*	*curvisetus*		MMETSP0716, MMETSP0717, MMETSP0718, MMETSP0719	CAMPEP_0187046754
	Ochrophyta	Bacillariophyceae	Chaetocerotace ae	*Chaetoceros*	*affinis*	CCMP159	MMETSP0088, MMETSP0090, MMETSP0091, MMETSP0092	CAMPEP_0187024338
	Ochrophyta	Bacillariophyceae	Corethraceae	*Corethron*	*pennatum*	L29A3	MMETSP0169, MMETSP0171	CAMPEP_0200351556, CAMPEP_0200360040

	Ochrophyta	Bacillariophyceae	Lithodesmiaceae	*Ditylum*	*brightwellii*	GSO103	MMETSP1002, MMETSP1005	CAMPEP_0187315252
	Ochrophyta	Bacillariophyceae	Lithodesmiaceae	*Ditylum*	*brightwellii*	GSO104	MMETSP1010, MMETSP1012, MMETSP1013	CAMPEP_0193980186
	Ochrophyta	Bacillariophyceae	Lithodesmiaceae	*Ditylum*	*brightwellii*	GSO105	MMETSP0998, MMETSP1001	CAMPEP_0187336034
	Ochrophyta	Bacillariophyceae	Cymatosiraceae	*Extubocellulus*	*spinifer*	CCMP396	MMETSP0699, MMETSP0697, MMETSP0698,	CAMPEP_0200501572
	Ochrophyta	Bacillariophyceae	Thalassiosiraceae	*Thalassiosira*	*rotula*	CCMP3096	MMETSP0403, MMETSP0404	CAMPEP_0192952656
	Ochrophyta	Bacillariophyceae	Thalassiosiraceae	*Thalassiosira*	*oceanica*	CCMP1005	MMETSP0970, MMETSP0971, MMETSP0972, MMETSP0973	CAMPEP_0192905742, CAMPEP_0192933258
	Ochrophyta	Bacillariophyceae	Thalassiosiraceae	*Thalassiosira*	*weissflogii*	CCMP1010	MMETSP0898, MMETSP0899, MMETSP0900, MMETSP0901, MMETSP1407, MMETSP1408, MMETSP1405, MMETSP1406, MMETSP1415, MMETSP1416, MMETSP1417, MMETSP1418, MMETSP1419, MMETSP1420, MMETSP1421, MMETSP1422, MMETSP1409, MMETSP1410, MMETSP1411, MMETSP1412, MMETSP1413, MMETSP1414,	CAMPEP_0193043134
	Ochrophyta	Bacillariophyceae	Thalassiosiraceae	*Thalassiosira*	*antarctica*	CCMP982	MMETSP0902, MMETSP0903, MMETSP0904, MMETSP0905	CAMPEP_0200088110
	Ochrophyta	Bacillariophyceae	Thalassiosiraceae	*Thalassiosira*	*weissflogii*	CCMP1336	MMETSP0878, MMETSP0879, MMETSP0880, MMETSP0881	CAMPEP_0193083268

	Ochrophyta	Bacillariophyceae	Thalassiosiraceae	*Thalassiosira*	*rotula*	GSO102	MMETSP0910, MMETSP0911, MMETSP0912, MMETSP0913	CAMPEP_0192998562
	Ochrophyta	Bacillariophyceae	Thalassiosiraceae	*Thalassiosira*	*gravida*	GMp14c1	MMETSP0492, MMETSP0493, MMETSP0494	CAMPEP_0200699684
	Ochrophyta	Bacillariophyceae	Rhizosoleniaceae	*Proboscia*	*alata*	PI-D3	MMETSP0174, MMETSP0176	CAMPEP_0200159498
	Ochrophyta	Bacillariophyceae	Skeletonemaceae	*Skeletonema*	*marinoi*	skelA	MMETSP0920, MMETSP0918	CAMPEP_0192233268
	Ochrophyta	Bacillariophyceae	Skeletonemaceae	*Skeletonema*	*dohrnii*	SkelB	MMETSP0562, MMETSP0563	CAMPEP_0192137106
	Ochrophyta	Bacillariophyceae	Skeletonemaceae	*Skeletonema*	*menzelii*	CCMP793	MMETSP0603, MMETSP0604	CAMPEP_0192272568
	Ochrophyta	Bacillariophyceae	Fragilariaceae	*Asterionellopsis*	*glacialis*	CCMP134	MMETSP0705, MMETSP0706, MMETSP0707, MMETSP0708	CAMPEP_0199870378, CAMPEP_0199870466
	Ochrophyta	Bacillariophyceae	Thalassionemataceae	*Thalassionema*	*nitzschioides*	L26-B	MMETSP0156, MMETSP0158	CAMPEP_0200190772
	Ochrophyta	Bacillariophyceae	Thalassionemataceae	*Thalassiothrix*	*antarctica*	L6-D1	MMETSP0152, MMETSP0154	CAMPEP_0200962748
	Ochrophyta	Chrysophyceae	Synuraceae	*Paraphysomonas*	*Imperforata*	PA2	MMETSP0103, MMETSP0104	CAMPEP_0190482658

	Ochrophyta	Chrysophyceae	Dinobryaceae	*Dinobryon*	sp.	UTEXLB2267	MMETSP0019, MMETSP0020, MMETSP0812	CAMPEP_0187279028, CAMPEP_0187277876
	Ochrophyta	Chrysophyceae	Ochromonadaceae	*Ochromonas*	sp.	CCMP1393	MMETSP0004, MMETSP0005	CAMPEP_0190272254
	Ochrophyta	Dictyochophyceae	Pedinellaceae	*Pseudopedinella*	*elastica*	CCMP716	MMETSP1068, MMETSP1097	CAMPEP_0191451212, CAMPEP_0191405316
	Ochrophyta	Eustigmatophyceae	Eustigmataceae	*Nannochloropis*	*gaditana*	B-31	http://www.ncbi.nlm.nih.gov/genome/11691?genome_assembly_id=53301	EWM28830.1
	Ochrophyta	Pelagophyceae	Pelagomonodaceae	*Aureococcus*	*anophagefferens*	CCMP1850	MMETSP0914, MMETSP0915, MMETSP0916, MMETSP0917	CAMPEP_0186659954, CAMPEP_0186691924
	Ochrophyta	Pelagophyceae	Sarcionochrysidaceae	*Aureoumbra*	*lagunensis*	CCMP1510	MMETSP0890, MMETSP0891, MMETSP0892, MMETSP0893	
	Ochrophyta	Pelagophyceae	Pelagomonodaceae	*Pelagococcus*	*subviridis*	CCMP1429	MMETSP0882, MMETSP0883, MMETSP0884, MMETSP0885	CAMPEP_0190535384
	Ochrophyta	Pelagophyceae	Pelagomonodaceae	*Pelagomonas*	*calceolata*	CCMP1756	MMETSP0888, MMETSP0889, MMETSP0886, MMETSP0887	CAMPEP_0199675904
	Ochrophyta	Raphidophyceae	Chattonellaceae	*Chattonella*	*subsalsa*	CCMP2191	MMETSP0947, MMETSP0948, MMETSP0949, MMETSP0950	CAMPEP_0187151886
	Ochrophyta	Raphidophyceae	Chattonellaceae	*Heterosigma*	*akashiwo*	CCMP2393	MMETSP0292, MMETSP0294, MMETSP0295, MMETSP0296	
	Ochrophyta	Raphidophyceae	Chattonellaceae	*Heterosigma*	*akashiwo*	NB	MMETSP0416, MMETSP0414, MMETSP0415	CAMPEP_0200245898

	Ochrophyta	Raphidophyceae	Chattonellaceae	*Heterosigma*	*akashiwo*	CCMP3107	MMETSP0409, MMETSP0410, MMETSP0411	CAMPEP_0188619588
	Ochrophyta	Raphidophyceae	Chattonellaceae	*Heterosigma*	*akashiwo*	CCMP 452	MMETSP0894, MMETSP0895, MMETSP0896, MMETSP0897	CAMPEP_0188660716
	Ochrophyta	Xanthophyceae	Vaucheriaceae	*Vaucheria*	*litorea*	CCMP2940	MMETSP0945, MMETSP0946	CAMPEP_0199157760
	Labyrinthista	Labyrinthulea	Thraustochytriidae	*Aurantiochytrium*	*limacinum*	ATCCMYA-1381	MMETSP0959, MMETSP0960, MMETSP0961, MMETSP0958	CAMPEP_0186647428
	Labyrinthista	Labyrinthulea	Thraustochytriaceae	*Schizochytrium*	*aggregatum*	ATCC28209	MMETSP0962, MMETSP0963, MMETSP0964, MMETSP0965	
**ALVEOLATA**	Dinoflagellata	Dinophyceae	Gymnodiniaceae	*Amphidinium*	*carterae*	CCMP1314	MMETSP0399, MMETSP0259, MMETSP0258, MMETSP0398C	CAMPEP_0186428018, CAMPEP_0186426112
	Dinoflagellata	Dinophyceae	Gymnodiniaceae	*Karenia*	*brevis*	CCMP2229	MMETSP0027, MMETSP0029, MMETSP0030, MMETSP0031	CAMPEP_0188846322, CAMPEP_0188912736
	Dinoflagellata	Dinophyceae	Gymnodiniaceae	*Karenia*	*brevis*	Wilson	MMETSP0202, MMETSP0201, MMETSP0648, MMETSP0649	CAMPEP_0189492014, CAMPEP_0189388456
	Dinoflagellata	Dinophyceae	Gymnodiniaceae	*Karenia*	*brevis*	SP3	MMETSP0527, MMETSP0528	CAMPEP_0189309980, CAMPEP_0189259884
	Dinoflagellata	Dinophyceae	Gymnodiniaceae	*Karenia*	*brevis*	SP1	MMETSP0573, MMETSP0574	CAMPEP_0189168478, CAMPEP_0189051810
	Dinoflagellata	Dinophyceae	Gymnodiniaceae	*Karlodinium*	*micrum*	CCMP2283	MMETSP1015, MMETSP1016, MMETSP1017	CAMPEP_0200794146, CAMPEP_0200809940,

								CAMPEP_0200812546, CAMPEP_0200785114
	Dinoflagellata	Dinophyceae	Peridiniaceae	*Durinskia*	*baltica*	CSIRO CS-38	MMETSP0117, MMETSP0116	CAMPEP_0200004706, CAMPEP_0200061890, CAMPEP_0199929530
	Dinoflagellata	Dinophyceae	Peridiniaceae	*Glenodinium*	*foliaceum*	CCAP 1116/3	MMETSP0118, MMETSP0119	CAMPEP_0188311894, CAMPEP_0188271626, CAMPEP_0188383902, CAMPEP_0188247870, CAMPEP_0188407728
	Dinoflagellata	Dinophyceae	Peridiniaceae	*Kryptoperidinium*	*foliaceum*	CCMP 1326	MMETSP0121, MMETSP0120	CAMPEP_0189696278, CAMPEP_0189642352, CAMPEP_0189900696, CAMPEP_0189771244
	Dinoflagellata	Dinophyceae	Peridiniaceae	*Peridinium*	*aciculiferum*	PAER-2	MMETSP0370, MMETSP0371	CAMPEP_0190670536, CAMPEP_0190612054,
	Dinoflagellata	Dinophyceae	Peridiniaceae	*Scrippsiella*	*trochoidea*	CCMP3099	MMETSP0270, MMETSP0271, MMETSP0272	CAMPEP_0191964902, CAMPEP_0191966966
	Dinoflagellata	Dinophyceae	Peridiniaceae	*Scrippsiella*	*Hangoei*	SHTV-5	MMETSP0359, MMETSP0360, MMETSP0361	CAMPEP_0191865414, CAMPEP_0191802586, CAMPEP_0191847402

	Dinoflagellata	Dinophyceae	Peridiniaceae	*Scrippsiella*	*hangoei-like*	SHHI-4	MMETSP0367, MMETSP0368, MMETSP0369	CAMPEP_0199174568, CAMPEP_0199204792, CAMPEP_0199287414
	Dinoflagellata	Dinophyceae	Goniodomataceae	*Alexandrium*	*monilatum*	CCMP3105	MMETSP0095, MMETSP0096, MMETSP0097, MMETSP0093	CAMPEP_0200518424, CAMPEP_0200664972
	Dinoflagellata	Dinophyceae	Goniodomataceae	*Alexandrium*	*fundyense*	CCMP1719	MMETSP0196C, MMETSP0347	CAMPEP_0185978494
	Dinoflagellata	Dinophyceae	Goniodomataceae	*Alexandrium*	*tamarense*	CCMP1771	MMETSP0382, MMETSP0384, MMETSP0378, MMETSP0380	CAMPEP_0186347800, CAMPEP_0186194818
	Dinoflagellata	Dinophyceae	Unknown	*Azadinium*	*spinosum*	3D9	MMETSP1036, MMETSP1037, MMETSP1038	CAMPEP_0186738362, CAMPEP_0186849724
	Dinoflagellata	Dinophyceae	Ceratiaceae	*Ceratium*	*fusus*	PA161109	MMETSP1075, MMETSP1074	CAMPEP_0199470176, CAMPEP_0199448012
	Dinoflagellata	Dinophyceae	Crypthecodiniacea	*Crypthecodinium*	*cohnii*	Seligo	MMETSP0323, MMETSP0325, MMETSP0326, MMETSP0324	CAMPEP_0193873420, CAMPEP_0193920440
	Dinoflagellata	Dinophyceae	Gonyaulacaceae	*Lingulodinium*	*polyedra*	CCMP 1738	MMETSP1032, MMETSP1033, MMETSP1034, MMETSP1035	CAMPEP_0190033118, CAMPEP_0189981950
	Dinoflagellata	Dinophyceae	Oxyrrhinaceae	*Oxyrrhis*	*marina*	CCMP1795	MMETSP0452_2, MMETSP0451_2C	
	Dinoflagellata	Dinophyceae	Oxyrrhinaceae	*Oxyrrhis*	*marina*	Unknown	MMETSP0468, MMETSP0469, MMETSP0470, MMETSP0471	

	Dinoflagellata	Dinophyceae	Oxyrrhinaceae	*Oxyrrhis*	*marina*	LB1974	MMETSP1424, MMETSP1425, MMETSP1426	
	Dinoflagellata	Dinophyceae	Prorocentraceae	*Prorocentrum*	*minimum*	CCMP1329	MMETSP0053, MMETSP0055, MMETSP0057, MMETSP0056	CAMPEP_0190887074, CAMPEP_0190973906, CAMPEP_0190881924
	Dinoflagellata	Dinophyceae	Prorocentraceae	*Prorocentrum*	*minimum*	CCMP2233	MMETSP0267, MMETSP0268, MMETSP0269	CAMPEP_0191171648, CAMPEP_0191085234, CAMPEP_0191101348
	Dinoflagellata	Dinophyceae	Symbiodiniaceae	*Symbiodinium*	*kawagutii*	CCMP2468	MMETSP0132_2, MMETSP0133_2, MMETSP0134_2, MMETSP0135_2	
	Dinoflagellata	Dinophyceae	Symbiodiniaceae	*Symbiodinium*	sp.	CCMP2430	MMETSP1115, MMETSP1116, MMETSP1117	CAMPEP_0192483534
	Dinoflagellata	Dinophyceae	Symbiodiniaceae	*Symbiodinium*	sp.	Mp	MMETSP1122, MMETSP1123, MMETSP1124, MMETSP1125	CAMPEP_0192623488
	Dinoflagellata	Dinophyceae	Symbiodiniaceae	*Symbiodinium*	sp.	C1	MMETSP1367, MMETSP1369	CAMPEP_0199632616, CAMPEP_0199584150
	Dinoflagellata	Dinophyceae	Symbiodiniaceae	*Symbiodinium*	sp.	C15	MMETSP1370, MMETSP1371	CAMPEP_0192427984
	Perkinsozoa	Perkinsea	Perkinsidae	*Perkinsus*	*chesapeaki*	ATCC PRA-65	MMETSP0925, MMETSP0924C	
	Perkinsozoa	Perkinsea	Perkinsidae	*Perkinsus*	*marinus*	ATCC 50439	MMETSP0923, MMETSP0922	

Table S2 – List of organisms examined in this study with respective nucleic acid database identifiers and sequence identifiers found for NDP-β-L-Rha biosynthesis.

References

1. Nakano, Y., Suzuki, N., Yoshida, Y., Nezu, T., Yamashita, Y., and Koga, T. (2000) Thymidine Diphosphate-6-deoxy-L-*lyxo*-4-hexulose Reductase Synthesizing dTDP-6-deoxy-L-talose from *Actinobacillus actinomycetemcomitans. J. Biol. Chem.* **275**, 6806-6812.
2. Elling, L., Rupprath, C., Günther, N., Römer, U., Verseck, S., Weingarten, P., Dräger, G., Kirschning, A., and Piepersberg, W. (2005) An enzyme module system for the synthesis of dTDP-activated deoxysugars from dTMP and sucrose. *ChemBioChem*, **6**, 1423-1430.
3. Yoo, H. G., Kwon, S. Y., Karki, S., and Kwon, H. J. (2011) A new route to dTDP-6-deoxy-L-talose and dTDP-L-rhamnose: dTDP-L-rhamnose 4-epimerase in *Burkholderia thailandensis. Bioorganic Med. Chem. Lett.* **21**, 3914-3917.
4. Shibaev, V. N., Eliseeva, G. P., Kusov, Y. Y., Petrenko, V. A., Mishchenko, S. S., and Kochetkov, N. K. (1976) Synthesis of β-L-rhamnosyl pyrophosphate esters of thymidine, uridine, and 2′-deoxyuridine. *Russ. Chem. Bull.* **25**, 2405-2408.
5. Shibaev, V. N., Kusov, Y. Y., Troitskii, M. F., and Kochetkov, N. K. (1974) Chemistry of glycosyl phosphates and their derivatives. *Bull.Acad. Sci. USSR. Div. Chem. Sci.* **23**, 171-173.
6. Prihar, H. S., and Behrman, E. J. (1973) Chemical synthesis of β-L-fucopyranosyl phosphate and β-L-rhamnopyranosyl phosphate. *Biochemistry*, **12**, 997-1002.
7. Offen, W., Martinez-Fleites, C., Yang, M., Kiat-Lim, E., Davis, B. G., Tarling, C. A., Ford, C. M., Bowles, D. J., and Davies, G. J. (2006) Structure of a flavonoid glucosyltransferase reveals the basis for plant natural product modification. *EMBO J.* **25**, 1396-1405.
8. Zhao, Y., and Thorson, J. S. (1998) A methodological comparison: The advantage of phosphorimidates in expanding the sugar nucleotide repertoire. *J. Org. Chem.* **63**, 7568-7572.
9. Timmons, S. C., and Jakeman, D. L. (2008) Stereospecific synthesis of sugar-1-phosphates and their conversion to sugar nucleotides. *Carbohydr.Res.* **343**, 865-874.
10. Wagner, G. K., Pesnot, T., and Field, R. A. (2009) A survey of chemical methods for sugar-nucleotide synthesis. *Nat. Prod. Rep.* **26**, 1172-1194.
11. Meyers, C. L. F., and Borch, R. F. (2001) A novel method for the preparation of nucleoside diphosphates. *Org. Lett.* **3**, 3765-3768.
12. Martinez, V., Ingwers, M., Smith, J., Glushka, J., Yang, T., and Bar-Peled, M. (2012) Biosynthesis of UDP-4-keto-6-deoxyglucose and UDP-rhamnose in pathogenic fungi *Magnaporthe grisea* and *Botryotinia fuckeliana*. *J. Biol. Chem.* **287**, 879-892.
13. Barber, G. A., and Behrman, E. J. (1991) The synthesis and characterization of uridine 5′-(β-L-rhamnopyranosyl diphosphate) and its role in the enzymic synthesis of rutin. *Arch. Biochem. Biophys.* **288**, 239-242.
14. Sabesan, S., and Neira, S. (1992) Synthesis of glycosyl phosphates and azides. *Carbohydr. Res.* **223**, 169-185.
15. Bock, K., and Pedersen, C. (1974) A study of ^{13}CH coupling constants in hexopyranoses. *J. Chem. Soc. Perkin. Trans.* **2**, 293-299.
16. Duus, J. Ø., Gotfredsen, C. H., and Bock, K. (2000) Carbohydrate structural determination by NMR spectroscopy: modern methods and limitations. *Chem. Rev.* **100**, 4589-4614.

17. Singh, G., and Vankayalapati, H. (2001) Efficient stereocontrolled synthesis of C-glycosides using glycosyl donors substituted by propane 1, 3-diyl phosphate as the leaving group. *Tetrahedron: Asymmetry*, **12**, 1727-1735.
18. Roseman, S., Distler, J. J., Moffatt, J. G., and Khorana, H. G. (1961) Nucleoside polyphosphates. XI. 1 An improved general method for the synthesis of nucleotide coenzymes. Syntheses of uridine-5', cytidine-5'and guanosine-5'diphosphate derivatives. *J. Am. Chem. Soc.* **83**, 659-663.
19. Moffatt, J. G., and Khorana, H. G. (1961). Nucleoside Polyphosphates. X. 1 The Synthesis and Some Reactions of Nucleoside-5'Phosphoromorpholidates and Related Compounds. Improved Methods for the Preparation of Nucleoside-5'Polyphosphates. *J. Am. Chem. Soc.* **83**, 649-658.
20. Wittmann, V., and Wong, C. H. (1997) 1 H-Tetrazole as catalyst in phosphomorpholidate coupling reactions: efficient synthesis of GDP-fucose, GDP-mannose, and UDP-galactose. *J. Org. Chem.* **62**, 2144-2147.
21. Rokade, S. M., and Bhate, P. M. (2015) One-pot synthesis of per-*O*-acetylated hemiacetals from free sugars in a deep eutectic solvent. *Carbohydr. Res.* **416**, 21-23.
22. Turnock, D. C., and Ferguson, M. A. (2007) Sugar nucleotide pools of *Trypanosoma brucei*, *Trypanosoma cruzi*, and *Leishmania major*. *Eukaryot. cell*, **6**, 1450-1463.
23. Räbinä, J., Mäki, M., Savilahti, E. M., Järvinen, N., Penttilä, L., and Renkonen, R. (2001) Analysis of nucleotide sugars from cell lysates by ion-pair solid-phase extraction and reversed-phase high-performance liquid chromatography. *Glycoconj. J.* **18**, 799-805.
24. Behmüller, R., Forstenlehner, I. C., Tenhaken, R., and Huber, C. G. (2014) Quantitative HPLC-MS analysis of nucleotide sugars in plant cells following off-line SPE sample preparation. *Anal. Bioanal. Chem.* **406**, 3229-3237.
25. Pabst, M., Grass, J., Fischl, R., Léonard, R., Jin, C., Hinterkörner, G., Borth, N., and Altmann, F. (2010) Nucleotide and nucleotide sugar analysis by liquid chromatography-electrospray ionization-mass spectrometry on surface-conditioned porous graphitic carbon. *Anal. Chem.* **82**, 9782-9788.
